# Burden and risk factors of chronic obstructive pulmonary disease in Sub-Saharan African countries, 1990–2019: a systematic analysis for the Global Burden of disease study 2019

**DOI:** 10.1016/j.eclinm.2023.102215

**Published:** 2023-10-02

**Authors:** Mulubirhan Assefa Alemayohu, Maria Elisabetta Zanolin, Lucia Cazzoletti, Peter Nyasulu, Vanessa Garcia-Larsen, GBD 2019 Sub-Saharan COPD Collaborators, Mulubirhan Assefa Alemayohu, Maria Eisabetta Zanolin, Lucia Cazzoletti, Peter S. Nyasulu, Vanessa Garcia-Larsen, Yonas Derso Abtew, Denberu Eshetie Adane, Miracle Ayomikun Adesina, Amadou Barrow, Alemshet Yirga Berhie, Belay Boda Abule Bodicha, Gashaw Sisay Chanie, Feleke Mekonnen Demeke, Diriba Dereje, Lankamo Ena Digesa, Michael Ekholuenetale, Daniel Berhanie Enyew, Hawi Leul Esayas, Adeniyi Francis Fagbamigbe, Getahun Fetensa, Kahsu Gebrekirstos Gebrekidan, Yibeltal Yismaw Gela, Habtamu Alganeh Guadie, Segun Emmanuel Ibitoye, Olayinka Stephen Ilesanmi, Tesfaye K. Kanko, Patrick D.M.C. Katoto, Samson Mideksa Legesse, Shafiu Mohammed, Jobert Richie Nansseu, Ogochukwu Janet Nzoputam, Chimezie Igwegbe Nzoputam, Oluwakemi Ololade Odukoya, Osaretin Christabel Okonji, Mayowa O. Owolabi, Bereket Beyene Shashamo, Yonatan Solomon, Worku Animaw Temesgen, Gedif Ashebir Wubetie, Yazachew Yismaw, Getachew Assefa Zenebe

**Affiliations:** a Unit of Epidemiology and Medical Statistics, University of Verona, Italy; b School Public Health, Mekelle University, Ethiopia; c Biostatistics and Clinical Epidemiology Unit, Department of Public Health, Experimental and Forensic Medicin, University of Pavia, 27100, Pavia, Italy; d Faculty of Medicine and Health Sciences, Stellenbosch University, Cape Town, South Africa; e Department of International Health, The Johns Hopkins Bloomberg School of Public Health, Baltimore, USA

**Keywords:** COPD, Chronic respiratory diseases, Burden, Global burden of disease, Risk factors, DALYs

## Abstract

**Background:**

Sub-Saharan Africa (SSA) has experienced a surge of non-communicable diseases (NCDs) including chronic obstructive pulmonary disease (COPD) over the past two decades. Using data from the Global Burden of Diseases, Injuries, and Risk Factors Study (GBD), in this study we have estimated the burden and attributable risk factors of COPD across SSA countries between 1990 and 2019.

**Methods:**

COPD burden and its attributable risk factors were estimated using data from the 2019 GBD. Percentage change was estimated to show the trend of COPD estimates from 1990 to 2019. COPD estimates attributable by risk factors were also reported to ascertain the risk factor that brings the greatest burden by sex and locations (at country and regions level).

**Findings:**

In 2019, all-age prevalent cases of COPD in SSA were estimated to be 10.3 million (95% Uncertainty Intervals (UI) 9.7 million to 10.9 million) showing an increase of 117% compared with the number of all-age COPD cases in 1990. From 1990 to 2019, SSA underwent an increased percentage change in all-age YLDs due to COPD ranging from 41% in Lesotho to 203% in Equatorial Guinea. The largest premature mortality due to COPD was reported from Central SSA accounting for 729 subjects (95% UI, 509–1078). The highest rate of DALYs attributable to COPD was observed in Lesotho. Household air pollution from solid fuel was the primary contributor of the age standardized YLDs, death rate, and DALYs rate per 100,000 population.

**Interpretation:**

The prevalence of COPD in SSA has had a steady increase over the past three decades and has progressively become a major public health burden across the region. Household air pollution from solid fuel is the primary contributor to COPD related burden, and its percentage contribution showed a similar trend to the reduction of COPD attributed age-standardized DALY rate. The methodological limitations of surveys and datapoints included in the GBD need to be considered when interpreting these associations.

**Funding:**

There are no specific fundings received for this study. The Global Burden of Disease study was supported by funding from the 10.13039/100000865Bill & Melinda Gates Foundation.

**Research in context**

**Evidence before this study**

Studies on COPD in SSA have increased in recent years, and the burden of COPD has been estimated by countries, international initiatives such as the BOLD and FRESH AIR, WHO, and the Global Burden of Disease Study (GBD). We conducted a systematic search in PubMed and Scopus with the term Pulmonary Disease, Chronic Obstructive [Mesh] AND (“Prevalence” OR “Mortality”) AND (“Sub-Saharan Africa” OR “Sub-Saharan Africa” OR “Sub-Saharan African countries” without any language restriction, for articles published up to January 20, 2022. We found studies including meta-analyses conducted from 2017 to 2020 reporting a pooled COPD prevalence of 8% [6%–11%] in SSA. In most of these studies, lack of consensus on case definition, missing data management, limited sample size, national and subnational study coverage, and standardization complicate hardly the estimation of COPD burden and its current status in Sub-Saharan African countries. Although the GBD 2019 capstone included COPD prevalence, mortality, and DALYs in SSA, no further details on COPD burden and risk factors were highlighted, nor comparisons were made between countries and subregions in SSA.

**Added value of this study**

This is the first large-scale report on the COPD burden in SSA based on GBD data. In comparison to previous studies including meta-analysis in SSA, our study reported more COPD parameters, such as prevalence, non-fatal health loss, mortality, and DALYs for chronic obstructive pulmonary disease and attributable risk factors. As part of GBD 2019, our study provides additional information on the burden and risk factors of COPD in SSA and also examines the observed changes in the period between 1990 and 2019 by sex and age across countries and subregions. All-age COPD prevalence increased by 117% in SSA, with a 21% decrease in age-standardized death rate, and COPD was responsible for 715 DALYs per 100,000 in SSA. Countries that invested in reducing household air pollution from solid fuels and improving their UHC system showed a significant reduction in the burden of COPD, while in contrast, countries with increased tobacco use showed an increase in the prevalence of COPD. Interestingly, household air pollution from solid fuels was found to be the main contributor in SSA, in contrast to the global perspective where smoking was the primary risk factor.

**Implications of all the available evidence**

Our study provides updated evidence on the burden of morbidity and premature mortality attributed to COPD in Sub-Saharan countries and regions from 1990 to 2019. Trends in COPD and the contribution of selected risk factors over the past three decades we found are an important move in improving our understanding of on the status and trend of COPD in Africa. This study can help to inform about the heterogeneity of burden and risk factors by country, as well as suggested interventions to reverse the COPD curve in SSA. In addition, our study shows a heterogeneous distribution of COPD and of the risk factor responsible for a large fraction of burden among countries in the region; as a result, intervention strategies can vary significantly from country to country, depending on the main risk factors responsible for most of the burden. The inherent limitations of the GBD data sources need to be considered when examining risk factors, as there are still limited surveys and data points available. Future research could be conducted to examine the role of other important environmental and lifestyle risk factors that could further explain the burden of COPD.

## Introduction

Globally, non-communicable diseases (NCDs) represent the biggest threats to health and development globally, particularly in developing countries^1^ accounting for more than 73% of the total deaths in 2017.^1^^,^^2^ Chronic respiratory diseases are the third major contributor of deaths^2^ accounted for 454 million people worldwide in 2019, of which 212 million (46.7%)^3^ were COPD cases. Regions with a low socio-demographic index were reported to have the greatest burden of chronic respiratory diseases.^4^^,^^5^ Current evidence suggests that Sub-Saharan African countries are at the dawn of the epidemiological transition^6^ and an increase of 31.5% of chronic obstructive pulmonary disease (COPD) cases over a decade that is attributable to ageing alone.^7^ Available evidence shows that COPD stage is associated with a high mortality, accounting for 95.90 deaths/1000 person-years in Uganda^8^ and stage 4 disease at baseline was the strongest risk factor associated with death.^8^^,^^9^ Despite the steady increase in the number of COPD cases, there are few studies collecting population-based data on COPD in Sub-Saharan African (SSA) countries.^10^, ^11^, ^12^

Epidemiological studies reported that the prevalence of COPD in SSA countries shows large variations, ranging from 1.1% to 23.8%.^7^^,^^12^, ^13^, ^14^, ^15^, ^16^, ^17^, ^18^, ^19^, ^20^, ^21^ Some of the variation is due to differences in the definitions and criteria used to ascertain the prevalence of spirometrically-defined COPD.^18^ In a prospective cross-sectional study conducted by van Gemert et al. in Uganda,^17^ COPD prevalence was 16.2% or 12.4%, depending on whether it was defined by the American Thoracic Society/European Respiratory Society (ATS/ERS), or Global initiative for chronic obstructive lung disease (GOLD) guidelines, respectively. Similarly, the mean age of COPD patients was estimated about 55.5 or 46.7 years, when using the ATS/ERS and GOLD guidelines, respectively.^17^ Another study conducted by North et al. in Uganda,^15^ however, found that prevalence of COPD was 2%, which was unchanged when defining COPD as FEV_1_/FVC<0.7.

The variation of COPD prevalence by age was observed on these epidemiological surveys using the GOLD^14^^,^^16^ and showed a six-fold higher prevalence in old compare to young age group,^16^ except in the study conducted by Magitta et al.^19^ A history of prior active TB,^16^^,^^19^ smoking,^14^^,^^17^^,^^19^ biomass exposure,^13^^,^^14^^,^^16^ poor ventilated kitchen^14^ were associated with the risk of COPD. The correlation between age and years of exposure to biomass smoke, and hours per day exposure to biomass smoke was found in subjects with COPD than without COPD.^14^

Lack of generalizability,^8^^,^^14^^,^^16^^,^^20^ limited sample size,^8^^,^^9^^,^^19^^,^^20^ high non-participation rate,^9^^,^^13^^,^^19^^,^^20^ challenges to identify causal risk factors risk factors,^8^^,^^13^^,^^15^ over- and under-diagnosis^14^, ^15^, ^16^, ^17^^,^^19^^,^^21^^,^^22^ and self-reported age^14^^,^^17^ have been described as some of the major limitations to understand the distribution and risk factors for COPD in Africa. An estimated 98.3% and 81.4% of spirometrically defined COPD cases were undiagnosed in Ife, Nigeria and Cape Town, South Africa, respectively.^22^ Moreover, inconsistent diagnostic criteria and variable methods and methodological quality^18^ and the heterogeneity in spirometrically defined COPD within and/or between studies^13^, ^14^, ^15^, ^16^, ^17^^,^^20^ are some others major limitations. This poses additional challenge not only to the paucity of nationally representative population based studies,^7^^,^^18^^,^^22^ but also to the understanding of the current status and progress being made at country level.^23^

The Global Burden of Diseases, Injuries, and Risk Factors Study (GBD) provides a systematic scientific assessment of published, publicly available, and contributed data; and also use an analytical approach to standardize and resolve the problem of missing data.^3^ In SSA, however, analyses of the burden and attributable risk factors of COPD are limited. The primary objective of this GBD paper is to address this evidence gap, by estimating the mortality, prevalence, risk factors, and disability associated with COPD in SSA, and examining the variations observed in the period between 1990 and 2019.

## Methods

### Overview

SSA is amongst the seven super-regions where the Global Burden of Disease (GBD) estimate was produced. In this paper, as part of the GBD 2019 round, we have studied the GBD estimate of COPD in SSA, as classified by the World Bank. The methods applied in this paper are the same as those described for the GBD 2019, mentioned elsewhere.^3^^,^^24^^,^^25^ Additional methodological details are available as a supplementary appendix on the GBD 2019 Capstones: Diseases and Injuries,^3^ and Risk Factors.^24^

### Non-fatal estimation for COPD: prevalence and years lived with disability (YLDs)

The GOLD spirometry-based post-bronchodilation measurement (<0.7 FEV_1_/FVC) was used as case definition and severity grading for COPD.^26^ An alternative case definition was also used from GOLD Pre-bronchodilation, Lower Limit of Normal (LLN) Pre and Post bronchodilation, and European Respiratory Society (ERS) guidelines.^27^ Furthermore, the International Classification of diseases (ICD) versions 9 and 10 codes associated with COPD were enrolled as methods of evaluating whether individuals had COPD or not: the codes 491–492, and 496 from ICD-9, and J41, J42, J43, J44, and J47 from ICD-10 were included. Codes J40 and 490 (Bronchitis, not specified as acute or chronic) and J47 and 494 (Bronchiectasis) were excluded.

Prevalence, incidence, remission, and hospital claims data were the main data sources obtained either from systematic reviews or from data contributed by GBD collaborators. The availability of a spirometry-based measure was used as inclusion criteria for all the data. For Sub-Saharan Africa, five distinct datapoints were listed as input sources for COPD prevalence estimation in GBD's Global Health Data Exchange repository (Table S1). Of these input sources, the data of the Burden of Obstructive Lung Disease (BOLD)^28^ was the only population-based survey data source.

DisMod-MR version 2.1, an updated Bayesian meta-regression analytical tool, was used to estimate COPD prevalence by location, age, sex, and year. Prior to actual modelling, datapoint and bias adjustment were performed using; (1) age-sex and sex split to disaggregate data reported not disaggregated into age and sex (2) a Meta-Regression—Bayesian, Regularized, Trimmed (MR-BRT) model for direct comparison of study designs and case definitions. The detailed procedures for both data and bias adjustment were well documented in a previous GBD paper.^3^

The prevalence of COPD was estimated in two steps. In the first step, the prevalence was calculated using the DisMod-MR 2.1 model. The model has been set to zero for remission, noting that individuals do not recover from COPD. Furthermore, the model setting also includes a series of country-level covariates assuming to apply Spatiotemporal Gaussian process regression (ST-GPR) for countries that were missing complete datasets. Healthcare Access and Quality index (HAQi), standardized exposure variables (SEV) for COPD, and the proportion of elevation over 1500 m were included as country-level covariates. In the second step, the GOLD class groupings in DisMod-MR 2.1 were used to estimate proportions of severity for COPD.

Following these two distinct steps, the Years Lived with Disability (YLDs) was estimated as a product of the prevalence of each sequela and the disability weight (DW) of the relative severity of the sequela on a scale between 0 and 1; i.e., 0 implies a state equivalent to full health, and 1, a state equivalent to death. Uncertainty was computed for YLDs considering uncertainty in prevalence and uncertainty in the DW. 1000 samples of the YLD distribution were generated using 1000 samples of comorbidity corrected by YLDs and 1000 samples of the DW assuming no correlation in the uncertainty in prevalence and DW. The supplementary appendix for 2019 GBD Capstone provides further methodological details on Nonfatal health outcome estimation.^3^

### COPD mortality estimation: death and years of life lost (YLLs)

Vital registration was the main data input source to derive the Causes of Death due to COPD using the standard CODEm modelling approach. Datapoints with high or low or implausible values, or with substantial conflict from established age or temporal patterns, or with substantial conflict with other data sources but from the same locations or locations with similar Socio-demographic Index were excluded as outliers.

Potential covariates were included in the model by their influence either as a positive standardized beta (increased death) or a negative (decreased death) standardized beta. A scalar of summary exposure to risk for COPD, smoking prevalence, total number of cigarettes smoked (5, 10, & 20 years), indoor air pollution, and the proportion of elevation over 1500 m were covariates with a positive standardized beta. On the other way, healthcare access and quality index, socio-demographic index, 10-year income per capita, and education were covariates with a negative standardized beta.

The death estimation adjustment was performed by CoDCorrect using unadjusted death estimates from COPD, the overall sum of death estimates from chronic respiratory diseases, and all-cause deaths derived from demographic estimation. Years of life lost (YLLs) due to COPD was calculated by multiplying the deaths due to COPD by the remaining life expectancy in GBD's standard life table based on the lowest observed mortality rate at each stage in any population over five million people. Further methodological details on the cause of death estimation was explained in the methods appendix for GBD 2019 capstone.^3^

### DALYs estimation

Disability-adjusted life year (DALYs) was obtained by adding YLLs and YLDs for each age-sex-location. Uncertainty was estimated by assuming the uncertainty in YLLs to be independent of uncertainty in YLDs. A samples of 1000 DALYs were generated using the sum of 1000 samples for YLLs and YLD. The estimation was computed by recalculating every outcome of interest 1000 times, drawing from distribution of the sampling error around input data, correction for measurement error, and estimates of residual non-sampling error and, model selection in case of cause of death estimate. The 95% UIs were computed by using the 2.5th and 97.5th ordered draw of the DALY uncertainty distribution.^3^

### Risk factor attributable burden estimation

**Fig. 1:**
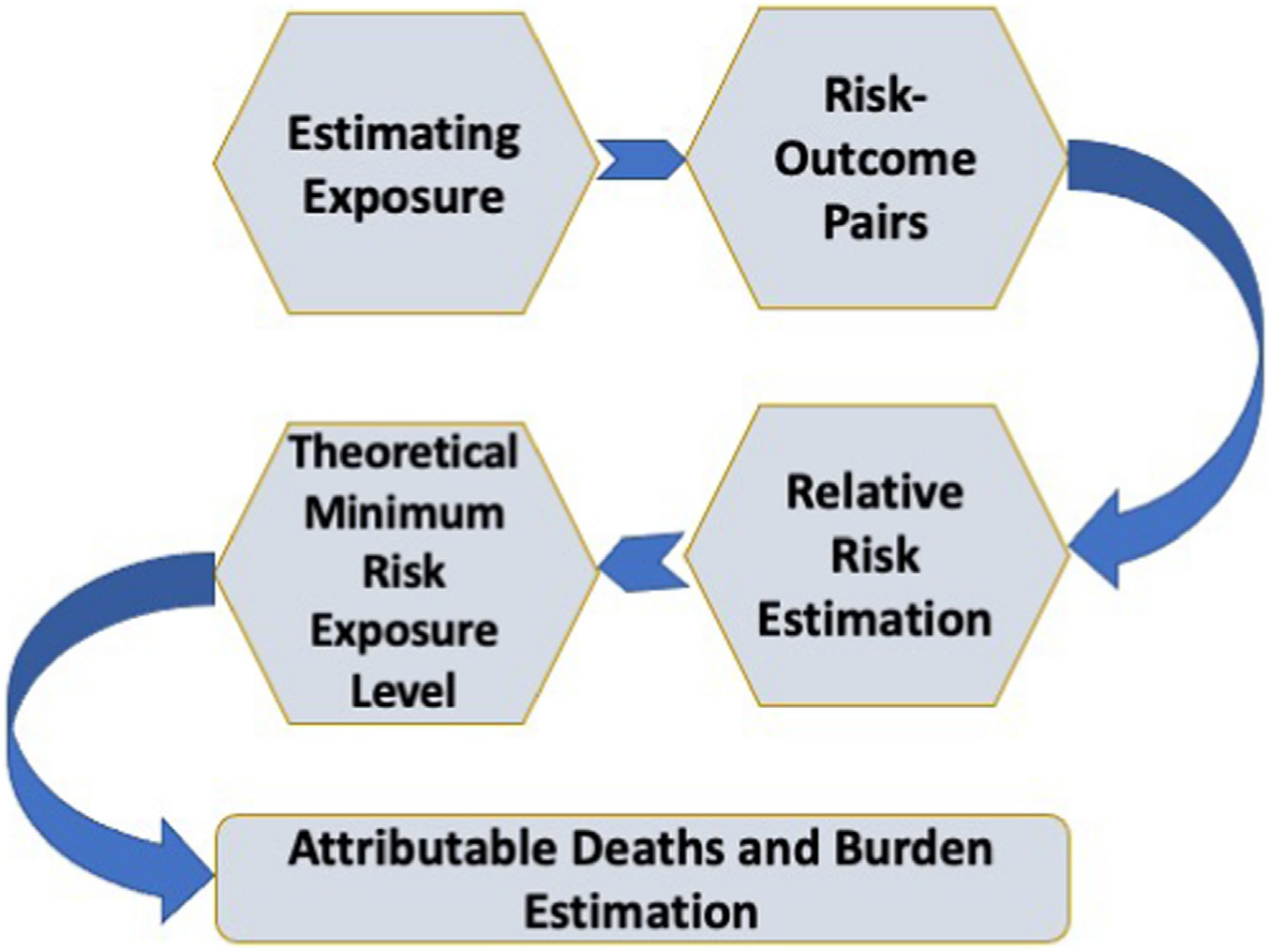
Flowchart shows risk factor attributable estimation using GBD comparative risk assessment framework.

Seven risk factors, namely, smoking, second-hand smoking, household air pollution from solid fuels, occupational particulate matter, ambient particulate matter, ozone, and low temperature were included in risk estimation for COPD. Those risk factors were included based on the World Cancer Research Fund criteria for convincing or probable evidence of risk–outcome pairs^29^ and the comparative risk assessment framework.^25^ Distinct datapoints for household air pollution from solid fuels (388), smoking (280), second-hand smoke (191), occupational particulate matter, gases, and fumes (81), ambient particulate matter pollution (24), and one datapoint for low temperature were available as exposure data input sources. There were no datapoint available for ambient ozone pollution exposure: it was, however, obtained through simulation modelling of ozone ground measurement data from the Tropospheric Ozone Assessment Report (TOAR). Population surveys were used as the main exposure data source for smoking, second-hand smoke, and household air pollution whereas the exposure to ambient PM2.5 was measured from satellite. Exposure data, relative risk of outcome, and a theoretical minimum level of exposure were used to estimate the population attributable fraction of COPD (Fig. 1). Further risk specific methodological details described in 2019 GBD risk factors capstone.^24^

### Data presentations

Findings were based on data obtained from GBD result tool.^30^ The reporting complies with the Guidelines for Accurate and Transparent Health Estimates Reporting (GATHER) statement.^31^ Global Burden of Disease (GBD) compare was used to explore the level and trend of estimates. In this study, COPD estimates (such as prevalence, YLDs, Deaths, YLLs, and DALYs) were reported for Sub-Saharan African countries, and four sub-regions (Central, Western, Eastern, and Southern). Those measures presented at country-level, group level (age or sex), change over time, and at-risk specific using the following metrics: number, rate, and percentage. Estimates were reported with their 95% uncertainty intervals (UI) when needed which were computed by ordering 1000 draws of a given estimate. Percentage change was estimated to show the trend of COPD estimates from 1990 to 2019. Furthermore, the percentage of COPD estimates attributable by risk factors was reported to highlight the risk factor that brought the greatest burden by sex at country and regional levels.

### Role of funding source

There is no role of funding sources in the study design; in the collection, analysis, and interpretation of data; in the writing of the report; and in the decision to submit the paper for publication.

## Results

### Nonfatal health loss attributable to COPD in Sub-Saharan Africa

**Fig. 2:**
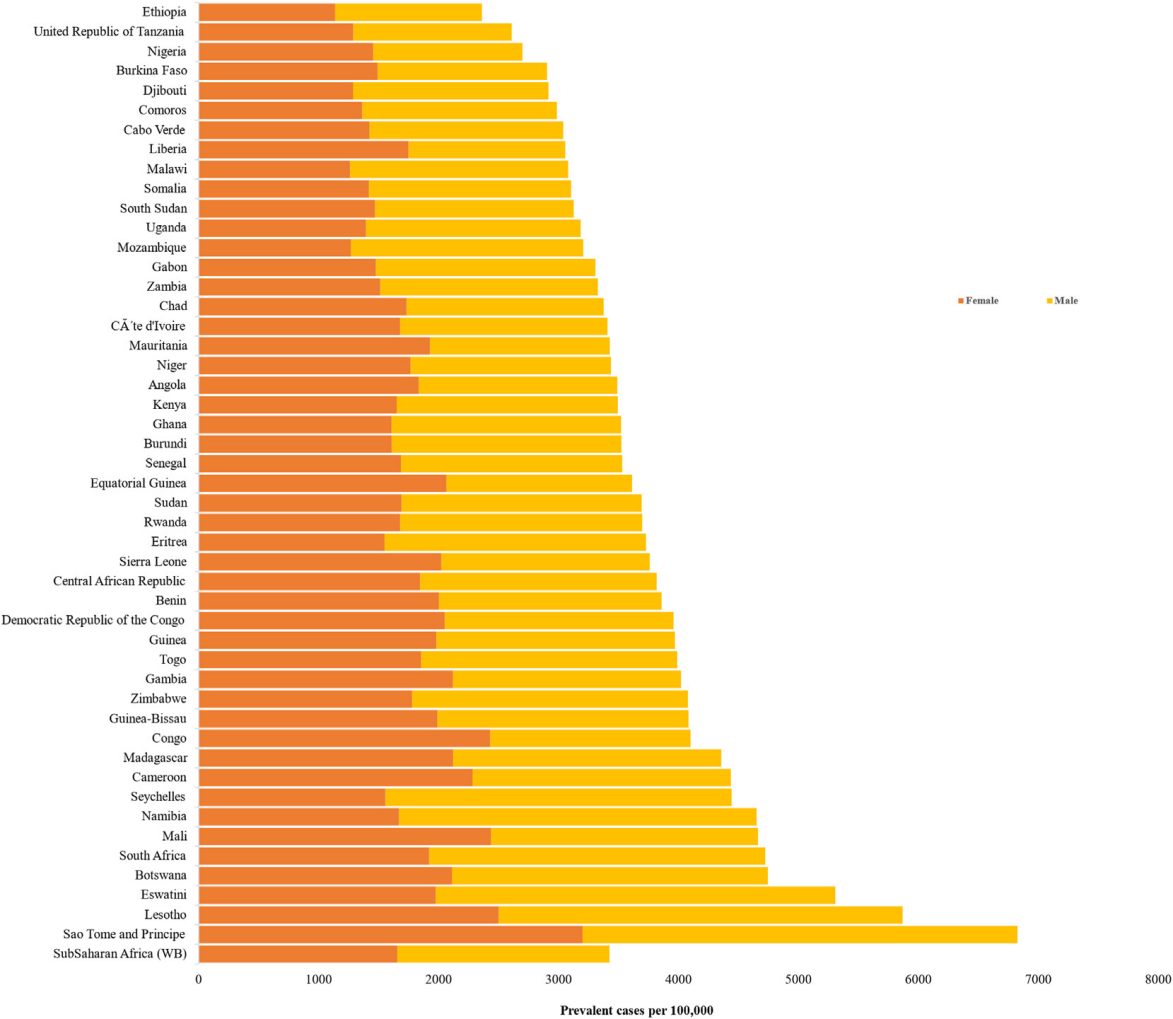
Age-standardized prevalence of COPD across Sub-Saharan African countries (World Bank Classification) in 2019.

In 2019, all-age prevalent cases of COPD in SSA were estimated to involve 10.3 million people (95% UI 9.7 million to 10.9 million), showing an increase of 117% compared with the number of all-age COPD cases in 1990 (Table 1). Chronic obstructive pulmonary disease (COPD) prevalence was estimated to be 1705 (95% UI 1820–1598) per 100,000, with differences by country and sex. Sao Tome and Principe, and Ethiopia had respectively the highest and lowest age-standardized COPD prevalence for both sexes [Fig. 2].

**Table 1:** Number and percentage change of all age COPD prevalence and COPD attributable YLDs in 2019 by Sub-Saharan Africa country.

	Number of prevalent cases in all-age, 2019 (95% UI)	Percentage change in all-age prevalent cases, 1990–2019	YLDs in all-age, 2019 (95% UI)	Percentage change in all-age YLDs, 1990–2019
Sub-Saharan Africa	10,340,971 (9,741,174–10,947,696)	117.0	1,264,690 (1,054,269–1,435,013)	117.1
Angola	254,081 (236,617–271,195)	189.2	31,766 (26,304–36,398)	191.1
Benin	122,253 (116,116–128,660)	162.8	14,729 (12,258–16,806)	165.0
Botswana	33,850 (31,750–36,001)	127.7	4463 (3711–5087)	129.5
Burkina Faso	180,238 (170,496–189,858)	148.8	21,904 (18,287–25,112)	150.7
Burundi	106,070 (99,945–112,433)	92.7	13,097 (10,804–15,002)	92.7
Côte d'Ivoire	248,534 (236,502–261,568)	141.1	29,846 (24,830–34,166)	143.6
Cape Verde	7318 (6951–7690)	78.0	901 (746–1015)	77.0
Cameroon	333,003 (315,180–351,183)	175.2	39,824 (33,428–45,642)	176.6
Central African Republic	52,811 (49,579–56,338)	92.6	6484 (5389–7440)	93.8
Chad	128,055 (121,772–134,579)	133.2	15,366 (12,941–17,510)	133.4
Comoros	8091 (7559–8638)	98.3	1003 (833–1152)	97.8
Congo	63,447 (59,272–67,413)	146.6	7891 (6520–9043)	147.3
Democratic Republic of the Congo	868,445 (808,523–929,199)	165.3	106,944 (89,130–122,971)	166.3
Equatorial Guinea	11,740 (10,799–12,645)	197.8	1462 (1207–1685)	203.0
Eritrea	67,089 (63,358–71,102)	161.3	8268 (6756–9485)	162.0
Eswatini	16,362 (15,605–17,119)	78.9	2111 (1770–2395)	74.2
Ethiopia	683,970 (612,886–751,405)	62.8	86,388 (70,077–101,068)	65.9
Gabon	19,732 (18,295–21,159)	93.9	2406 (1999–2768)	93.3
Gambia	24,182 (22,965–25,460)	170.4	2901 (2405–3312)	170.0
Ghana	353,063 (335,031–371,410)	193.9	40,968 (33,983–46,466)	193.8
Guinea	134,216 (126,884–141,604)	105.2	16,198 (13,509–18,503)	105.2
Guinea-Bissau	19,764 (18,753–20,857)	91.3	2378 (1972–2725)	92.8
Kenya	488,588 (452,563–528,500)	165.7	61,034 (50,552–70,346)	164.1
Lesotho	38,827 (37,057–40,680)	42.2	5043 (4196–5717)	41.5
Liberia	42,788 (40,388–45,276)	160.9	5110 (4267–5829)	162.2
Madagascar	304,017 (286,418–321,707)	147.7	37,925 (31,200–43,165)	149.3
Malawi	147,080 (137,355–156,496)	105.9	18,258 (15,192–20,946)	105.3
Mali	247,544 (232,568–261,553)	139.5	29,672 (24,863–33,898)	140.9
Mauritania	42,163 (39,788–44,643)	95.3	5101 (4293–5789)	95.9
Mauritius	32,393 (30,706–34,221)	83.0	3294 (2653–3799)	79.3
Mozambique	234,441 (220,557–247,596)	144.9	28,838 (23,886–32,900)	143.7
Namibia	33,664 (31,536–35,779)	78.8	4337 (3591–4966)	77.0
Niger	179,661 (169,575–189,387)	185.9	21,792 (18,106–24,915)	187.1
Nigeria	1,587,744 (1,449,908–1,718,545)	85.6	192,661 (158,614–222,305)	89.0
Rwanda	134,377 (126,021–143,261)	93.9	16,705 (13,834–19,161)	94.9
Sao Tome and Principe	3934 (3787–4087)	124.8	466 (389–523)	123.1
Senegal	159,836 (151,194–168,298)	130.2	19,073 (15,936–21,762)	130.5
Seychelles	2377 (2249–2512)	115.0	245 (199–282)	112.6
Sierra Leone	86,262 (81,988–90,499)	129.7	10,367 (8708–11,739)	130.8
Somalia	147,261 (136,640–157,532)	161.9	18,403 (15,194–21,312)	163.4
South Africa	1,046,594 (970,945–1,127,782)	76.9	135,801 (112,436–155,679)	73.8
South Sudan	79,130 (73,518–84,549)	58.1	9709 (8066–11,166)	59.9
Sudan	428,128 (401,529–455,381)	141.5	40,242 (32,532–46,905)	139.4
Togo	91,070 (86,074–96,044)	177.6	10,964 (9134–12,440)	177.5
Uganda	303,362 (278,483–326,245)	130.5	37,877 (30,979–43,760)	132.8
United Republic of Tanzania	423,197 (389,806–453,143)	141.4	53,252 (43,694–61,474)	142.9
Zambia	152,038 (141,443–161,813)	157.1	18,848 (15,572–21,698)	155.4
Zimbabwe	168,183 (157,789–179,080)	73.4	22,375 (18,389–25,608)	73.8

Notes: YLDs-years lived with disabilities. 95% UI-95% uncertainty intervals. COPD-chronic obstructive pulmonary disease.

**Fig. 3:**
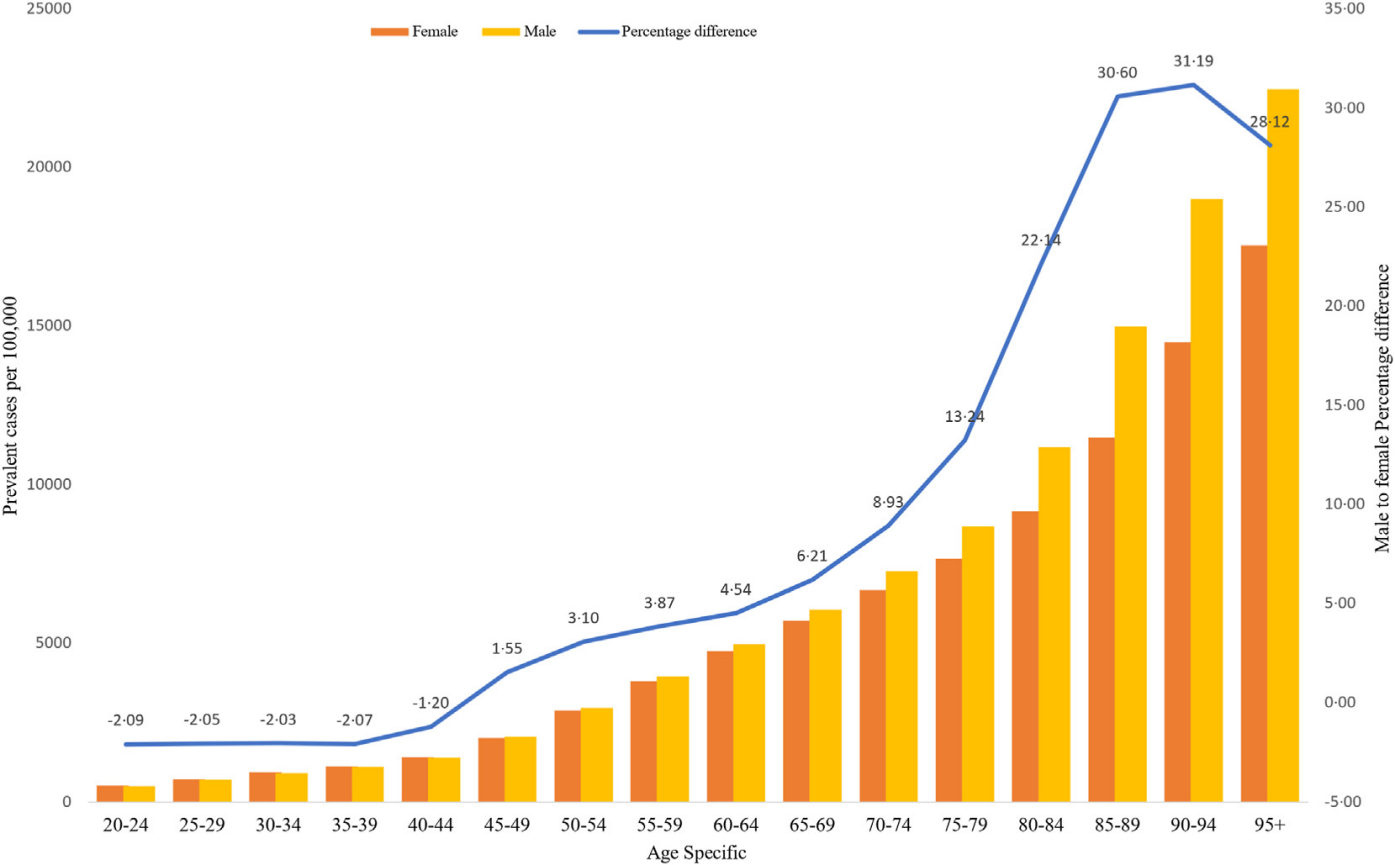
Age-specific prevalence of COPD by sex in Sub-Saharan Africa, 2019 (percentage difference was estimated to show percentage increase or decrease in male compared to female by age-specific).

**Fig. 4:**
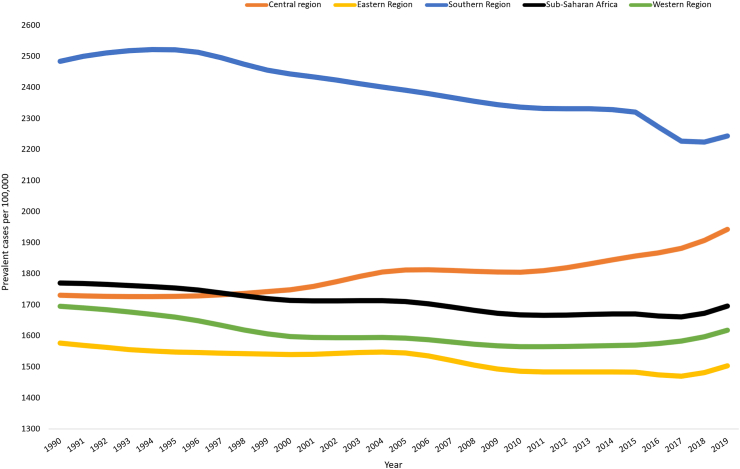
Trend in age-standardized prevalence rate of COPD by region in Sub-Saharan Africa, 1990–2019.

The age-specific prevalence of COPD started to increase at age 45 years for both sexes. Important percentage differences in COPD prevalence were observed between males and females from age 75 years ranging from 13% to 31% and reaching the peak at age 90–94 years [Fig. 3]. From 1990 to 2019 the prevalence rate of COPD was stable in Eastern and Western Sub-Saharan Africa, whereas there was a decreasing trend in Southern Sub-Saharan African countries. Central Sub-Saharan Africa countries showed an increase in age-standardized COPD prevalence since 2000 which reached a peak in 2019 [Fig. 4].

In 2019, Sub-Saharan Africa counted 1.2 millions (95% UI 1.1 millions to 1.4 millions) years lived with disabilities (YLDs) due to COPD. Nigeria, followed by South Africa and Democratic Republic Congo, had the highest YLDs due to COPD in 2019. From 1990 to 2019, Sub-Saharan African countries presented an increased percentage change in all-age YLDs due to COPD ranging from 41% in Lesotho to 203% in Equatorial Guinea (Table 1). Of Sub-Saharan countries, Ethiopia exhibited the highest reduction in percentage change of age standardized YLDs due to COPD among males from 1990 to 2019 accounting for 28.7%. Whereas in Ghana, the percentage change of age standardized YLDs due to COPD among males increased by 46.8% (Appendix Table A1).

**Fig. 5:**
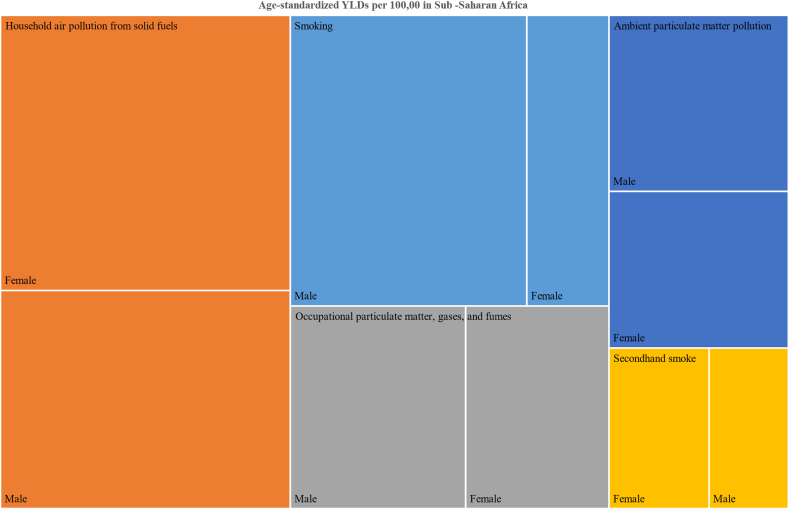
Age-standardized YLDs due to COPD attributable to risk factors by sex in sub–Saharan Africa in 2019 (the shaded area indicates the age-standardized years lived with disability rate attributable to the specific risk factor by sex).

Seven risk factors were considered based on the GBD comparative risk assessment framework. In 2019, household air pollution from solid fuel followed by smoking were the major contributors to the age standardized YLDs rate per 100,000 in Sub-Saharan Africa (Fig. 5). Smoking followed by ambient particulate matter pollution accounted for the major fraction of COPD attributable YLDs in southern Sub-Saharan Africa (Appendix Table A4). In Eastern Sub-Saharan Africa household air pollution from solid fuel was the major contributor to the percentage of COPD related YLDs among Females (51%), whereas smoking was responsible for the 8.7% (Appendix Table A4).

### Deaths and years life lost (YLLs) due to chronic obstructive pulmonary disease

**Fig. 6:**
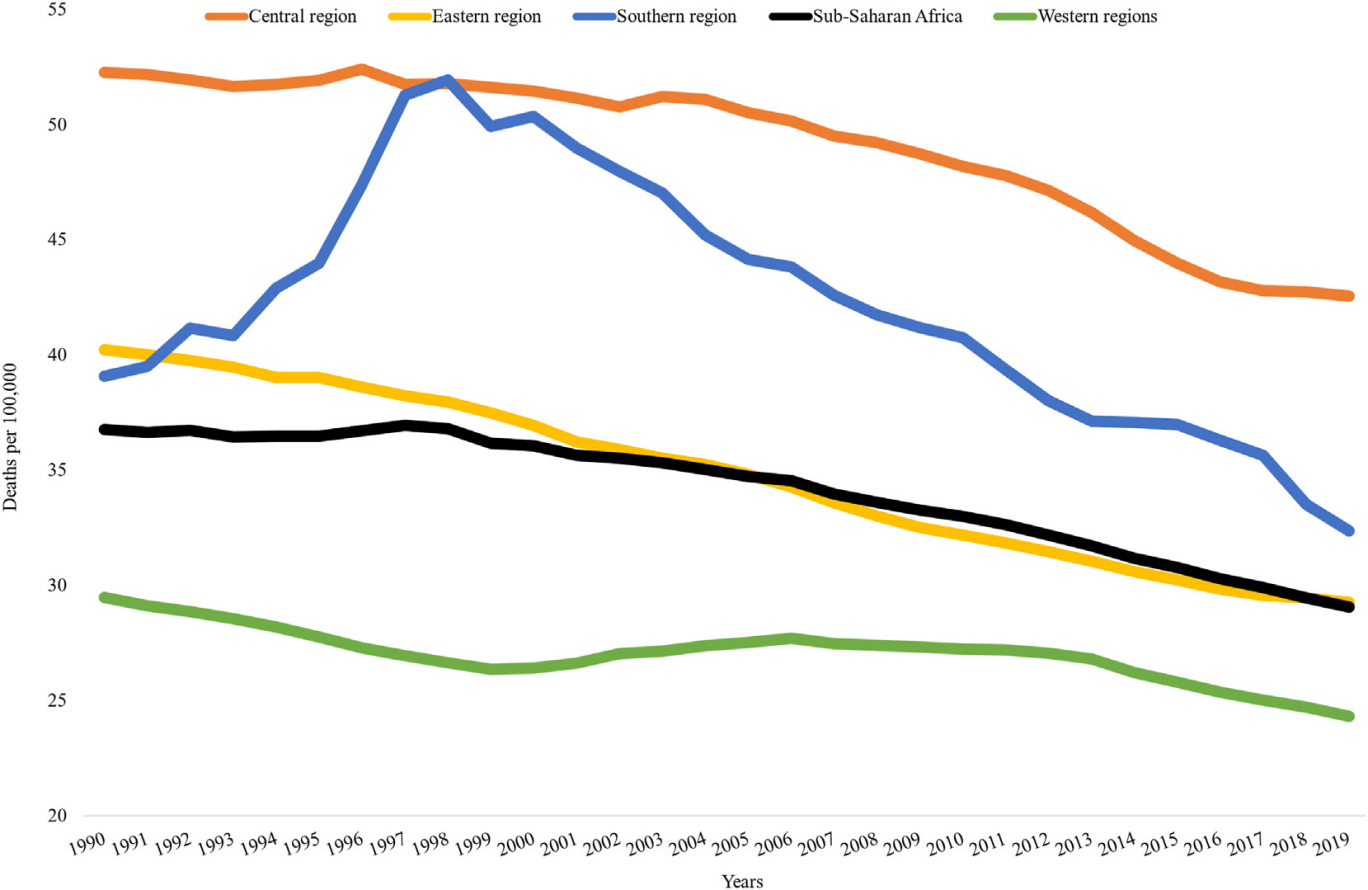
Trends in age standardized death due to COPD across Sub Saharan African regions, 1990–2019.

**Fig. 7:**
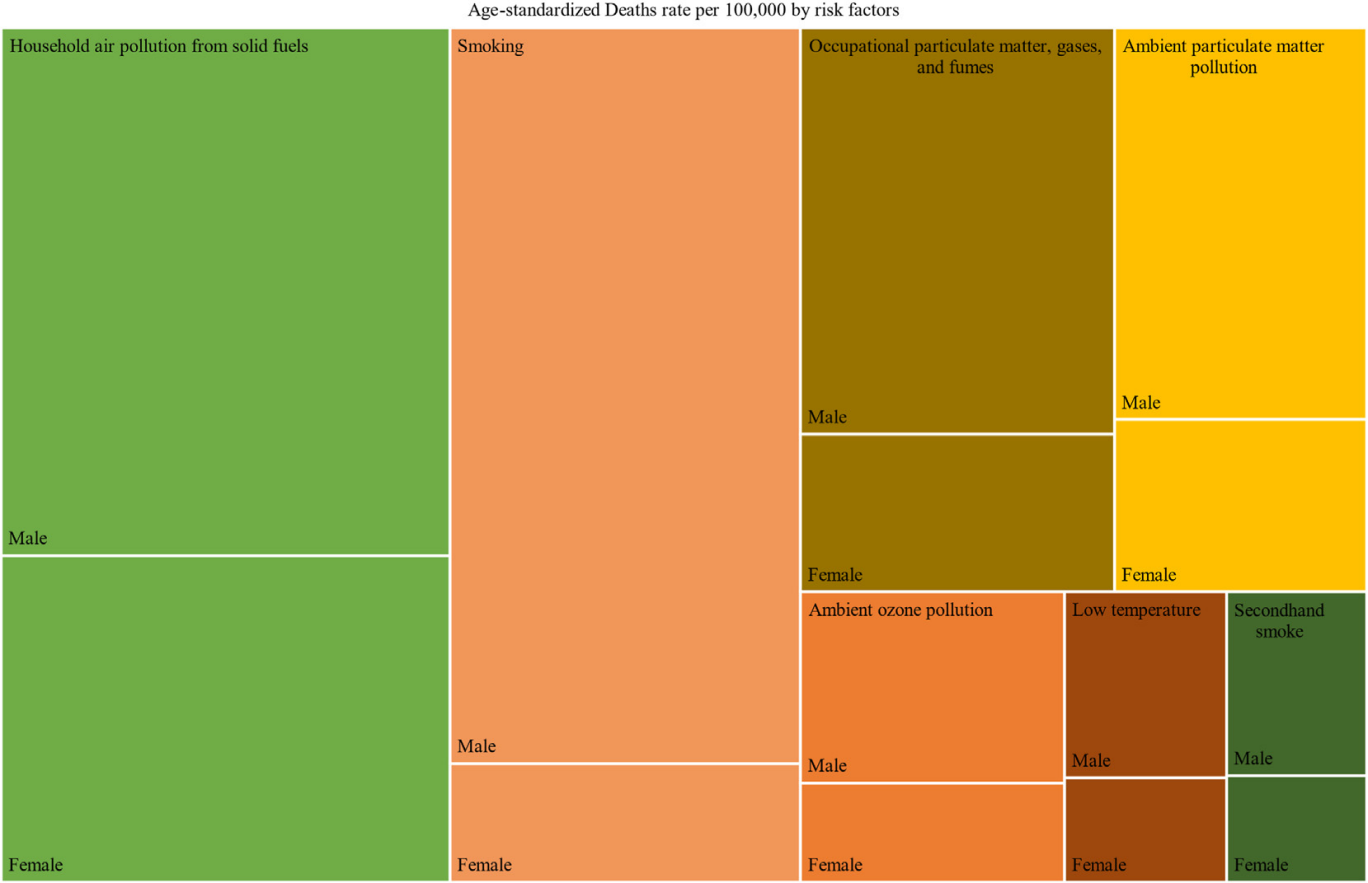
Contribution risk factors age-standardized Death rate due to COPD in 2019 (the shaded area indicates the age-standardized deaths rate attributable to the specific risk factor by sex).

In 2019, about 107 thousand people (95% UI 92 thousand to 123 thousand) died due to COPD in Sub-Saharan Africa, with a 21% decrease in age-standardized death rate compared to 1990. The highest reduction of COPD age-standardized death rate was reported in Cape Verde (48.5%) for both sexes (Appendix Table A2) followed by Rwanda (42.6%) and Equatorial Guinea (42.5%) in 2019 compared to the age standardized death rate in 1990. On other hand, only in Sao Tome and Principe COPD age standardized death rate increased by 1.5% in the same period (Table 2). Sub-Saharan African countries presented a reduction in COPD caused age-standardized YLL in females ranging from 0.8% in Lesotho to 55.0% in Cape Verde, from 1990 to 2019 (Appendix Table A2). From 1990 to 1998, Southern Sub-Saharan African exhibited a rapid increase in rate of deaths due to COPD and then a decrease since 1999 [Fig. 6]. In 2019, household air pollution followed by smoking and occupational particulate matters was responsible for the largest fraction of the age-standardized death and YLLs rate due to COPD in sub–Saharan Africa (Fig. 7, Appendix Table A5, Appendix Table A6).

**Table 2:** COPD attributable deaths and YLLs in 2019 and percentage change from 1990 to 2019 across Sub-Saharan Africa country.

	Number, all-age, 2019 (95% UI)		Age-standardized rate per 100,000 in 2019 (95% UI)		% Change in Age-standardized, 1990–2019	
	Deaths	YLLs	Deaths	YLLs	Deaths	YLLs
Sub-Saharan Africa	107,899 (92,406–123,565)	2,337,318 (1,967,700–2,722,757)	29 (25–33)	510 (436–587)	−21.0	−24.7
Angola	2245 (1673–2864)	50,767 (37,555–64,790)	31 (23–39)	515 (384–654)	−36.1	−41.8
Benin	1016 (779–1321)	22,788 (16,740–30,713)	26 (20–33)	464 (352–612)	−31.3	−32.3
Botswana	405 (276–540)	8893 (5780–12,182)	39 (27–52)	706 (477–947)	−30.4	−30.5
Burkina Faso	1391 (1122–1724)	33,244 (25,736–43,299)	19 (16–23)	350 (282–434)	−16.3	−14.9
Burundi	1464 (1100–1924)	34,901 (25,324–48,141)	43 (32–55)	784 (588–1033)	−29.6	−33.9
Côte d'Ivoire	1950 (1472–2498)	45,903 (33,350–60,748)	25 (20–31)	447 (335–574)	−33.3	−34.8
Cape Verde	73 (58–100)	1227 (1005–1681)	18 (14–25)	296 (243–403)	−48.5	−53.6
Cameroon	2311 (1583–3034)	53,612 (34,746–72,911)	25 (18–32)	453 (307–599)	−33.3	−32.8
Central African Republic	802 (535–1137)	20,399 (13,494–29,091)	53 (35–79)	1003 (669–1424)	−13.9	−15.7
Chad	1405 (1045–1831)	32,536 (24,055–43,439)	31 (23–40)	568 (417–741)	−14.2	−15.4
Comoros	109 (84–141)	2095 (1584–2750)	26 (20–33)	451 (347–588)	−33.5	−35.2
Congo	619 (449–800)	13,239 (9162–17,632)	35 (26–44)	579 (420–748)	−36.2	−41.6
Democratic Republic of the Congo	11,865 (7720–18,665)	257,781 (167,118–383,962)	47 (30–77)	801 (520–1251)	−12.7	−14.3
Equatorial Guinea	101 (65–164)	1995 (1247–3140)	30 (20–51)	474 (303–771)	−42.5	−52.1
Eritrea	637 (447–820)	15,980 (11,273–20,931)	33 (23–42)	621 (435–799)	−19.6	−26.4
Eswatini	192 (143–253)	4300 (3102–5765)	42 (31–55)	779 (572–1026)	−27.5	−27.1
Ethiopia	9309 (7480–10,922)	189,662 (150,989–229,253)	28 (23–33)	484 (386–573)	−38.9	−48.7
Gabon	204 (141–263)	4019 (2696–5350)	26 (18–33)	427 (295–560)	−35.5	−39.6
Gambia	244 (185–311)	5138 (3777–6655)	30 (23–38)	542 (405–697)	−11.4	−12.5
Ghana	3286 (1973–4138)	74,523 (44,339–94,611)	26 (16–32)	471 (281–592)	−11.3	−10.4
Guinea	1478 (1123–1863)	32,228 (24,009–41,774)	31 (24–39)	574 (432–733)	−15.1	−14.6
Guinea-Bissau	197 (144–256)	4991 (3537–6617)	34 (26–44)	671 (491–872)	−30.8	−33.1
Kenya	5081 (3957–6680)	111,390 (86,236–147,048)	31 (24–41)	542 (422–713)	−6.0	−4.3
Lesotho	712 (504–972)	15,846 (10,939–22,023)	71 (50–95)	1315 (921–1803)	−4.6	−2.6
Liberia	292 (213–405)	6481 (4562–9216)	18 (14–25)	325 (236–453)	−14.3	−19.3
Madagascar	3317 (2390–4440)	81,550 (58,547–109,571)	43 (31–56)	775 (557–1037)	−9.2	−12.2
Malawi	1450 (1148–1769)	31,668 (24,456–39,694)	25 (20–30)	449 (352–550)	−20.1	−21.4
Mali	2476 (1849–3175)	59,059 (41,107–79,482)	34 (26–42)	667 (482–866)	−11.1	−14.6
Mauritania	320 (246–405)	6098 (4478–8097)	19 (15–23)	308 (231–401)	−38.8	−45.6
Mauritius	293 (230–377)	4833 (3783–6263)	19 (15–25)	298 (234–385)	−38.4	−41.3
Mozambique	2182 (1662–2900)	50,412 (37,358–68,322)	25 (20–33)	471 (355–632)	−4.3	−2.8
Namibia	534 (403–686)	10,259 (7637–13,323)	45 (34–58)	777 (585–1008)	−28.4	−30.3
Niger	1790 (1281–2557)	43,149 (29,852–62,560)	31 (22–43)	547 (389–790)	−18.4	−23.7
Nigeria	14,120 (10,738–17,549)	286,800 (216,038–367,679)	22 (17–27)	362 (274–456)	−13.1	−19.0
Rwanda	1620 (1265–2093)	35,938 (27,489–46,777)	36 (28–46)	629 (490–816)	−42.6	−48.5
Sao Tome and Principe	48 (37–60)	996 (738–1244)	58 (44–72)	1014 (761–1261)	1.5	0.1
Senegal	1579 (1240–1948)	32,742 (24,919–42,053)	26 (20–31)	451 (350–567)	−27.9	−29.9
Seychelles	23 (19–27)	420 (338–491)	25 (21–29)	411 (335–479)	−23.6	−28.3
Sierra Leone	802 (591–1054)	17,996 (12,734–24,335)	27 (20–35)	494 (359–658)	−23.3	−23.2
Somalia	1991 (1263–3200)	51,568 (31,587–86,630)	40 (26–63)	771 (491–1219)	−23.4	−24.4
South Africa	11,843 (10,780–13,332)	225,719 (206,694–253,944)	32 (29–36)	536 (490–605)	−16.2	−19.3
South Sudan	757 (511–1048)	16,093 (10,751–23,267)	27 (18–36)	456 (305–638)	−26.5	−31.3
Sudan	4390 (2883–6190)	97,251 (62,467–139,562)	29 (19–40)	510 (332–725)	−21.5	−27.3
Togo	717 (527–942)	17,134 (12,156–23,224)	26 (20–34)	479 (350–633)	−25.9	−25.0
Uganda	3322 (2431–4316)	73,369 (51,430–98,685)	30 (22–39)	537 (390–704)	−31.6	−32.4
United Republic of Tanzania	4259 (3417–5100)	91,879 (72,713–113,177)	21 (17–25)	381 (300–458)	−19.3	−21.2
Zambia	1462 (1150–1793)	33,147 (25,578–41,889)	29 (23–35)	520 (407–638)	−19.5	−21.1
Zimbabwe	1217 (842–1582)	25,305 (16,898–33,012)	24 (17–31)	404 (279–525)	−9.5	−7.6

Notes: YLLs-years life lost; 95% UI-95% uncertainty intervals; COPD-chronic obstructive pulmonary disease; WB- World Bank.

### DALYs due to chronic obstructive pulmonary disease

**Fig. 8:**
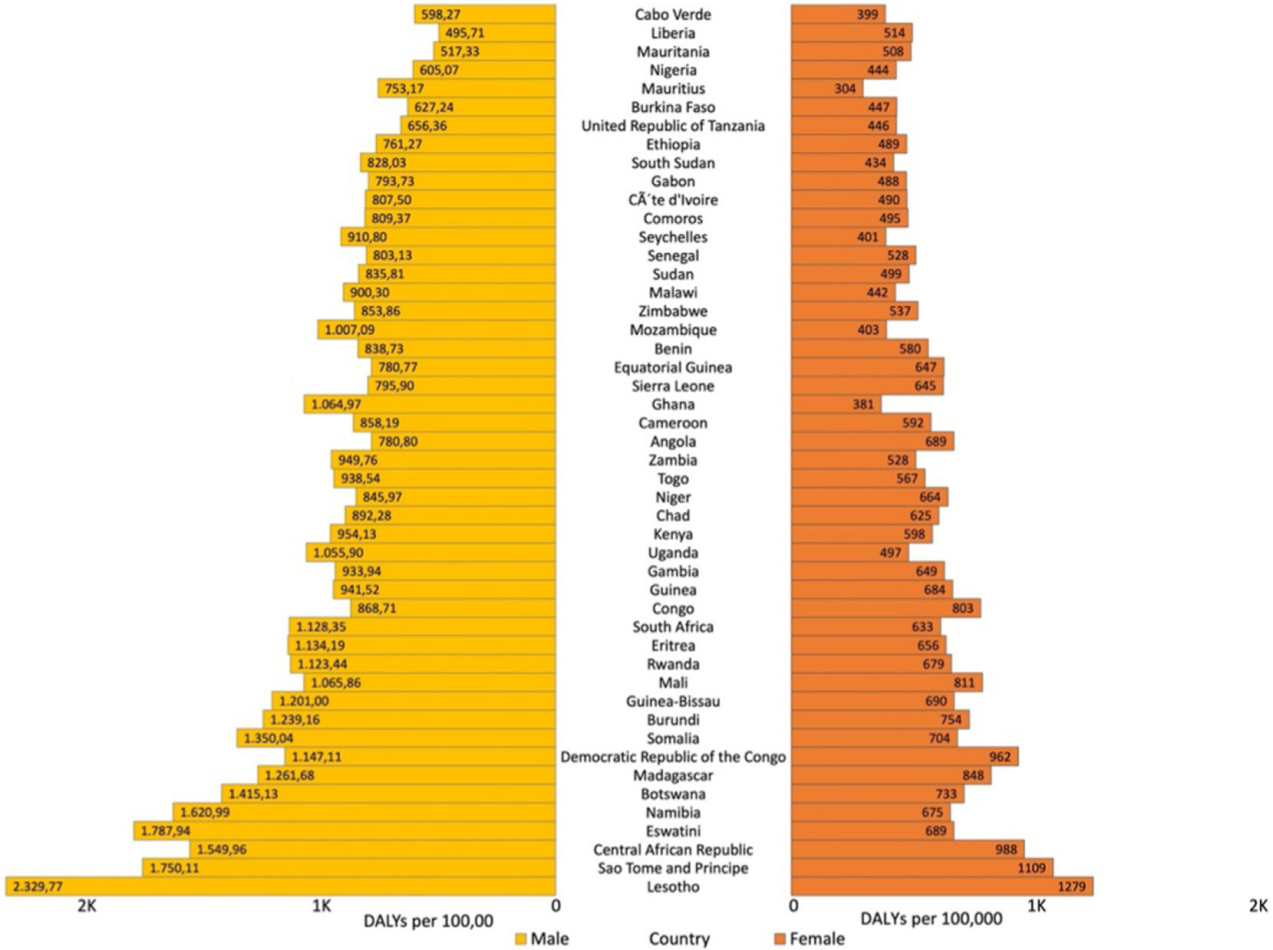
Age standardized DALYs due to COPD by sex across sub–Saharan Africa, 2019.

In 2019, COPD was responsible for 715 [880 in males, 577 in females] DALYs rate per 100,000 in Sub-Saharan Africa. Lesotho had the highest rate of DALYs attributable to COPD both for males [2329 per 100,000] and females [1279 per 100,000] whereas Liberia [495 per 100,000] and Mauritius [304 per 100,000] had the lowest rate of DALYs both for males and females, respectively [Fig. 8]. All Sub-Saharan countries except Mozambique, and Sao Tome and Principe showed a reduction in COPD related DALYs rate where Ethiopia and Ghana showed the highest and lowest percentage reduction, respectively. The percentage reduction in age standardized DALYs rate showed a variation by sex (Appendix Table A3).

**Fig. 9:**
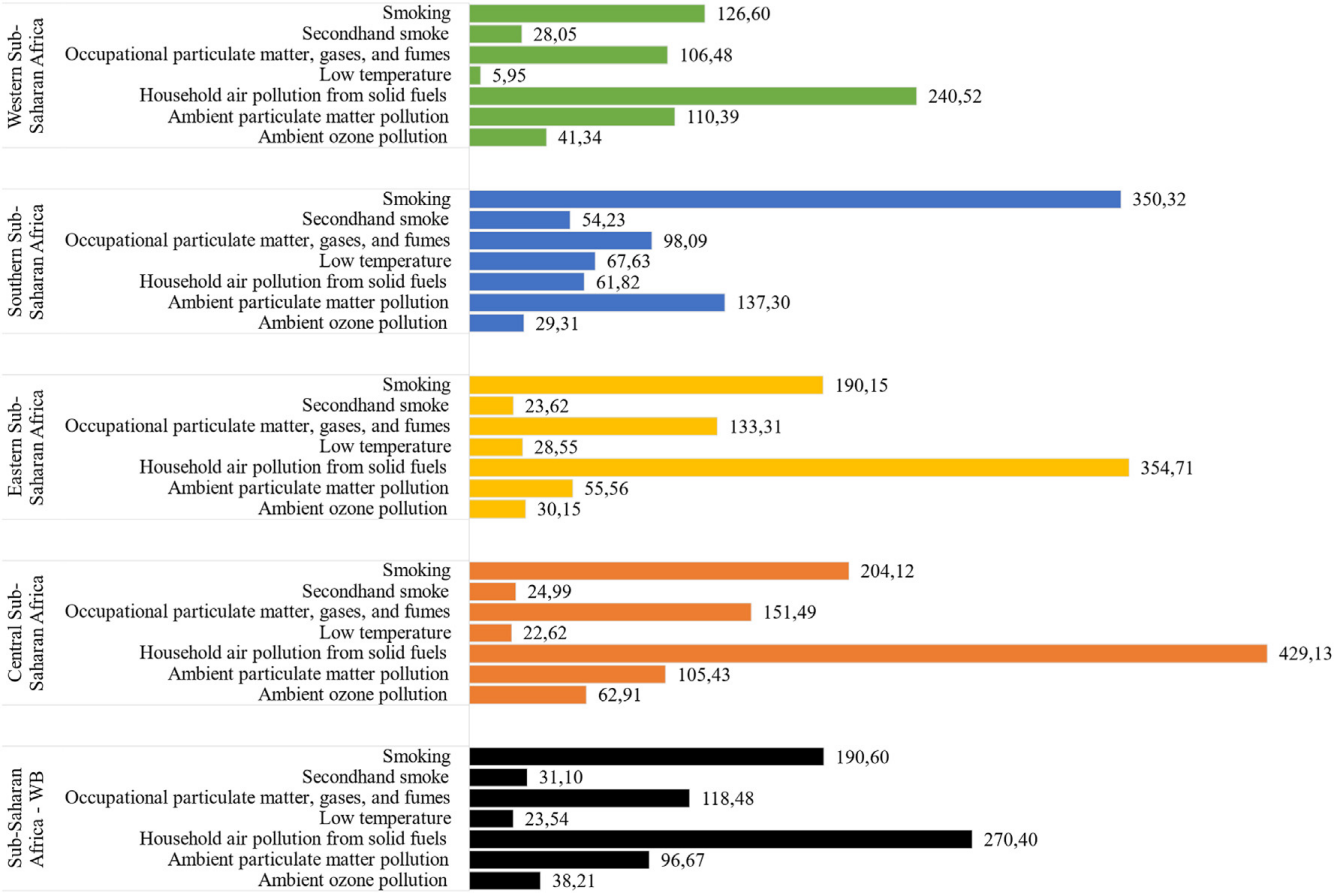
Age standardized DALYs rate (per 100,000) due to COPD attributable by risk factors among Sub-Saharan Africa regions in 2019.

**Fig. 10:**
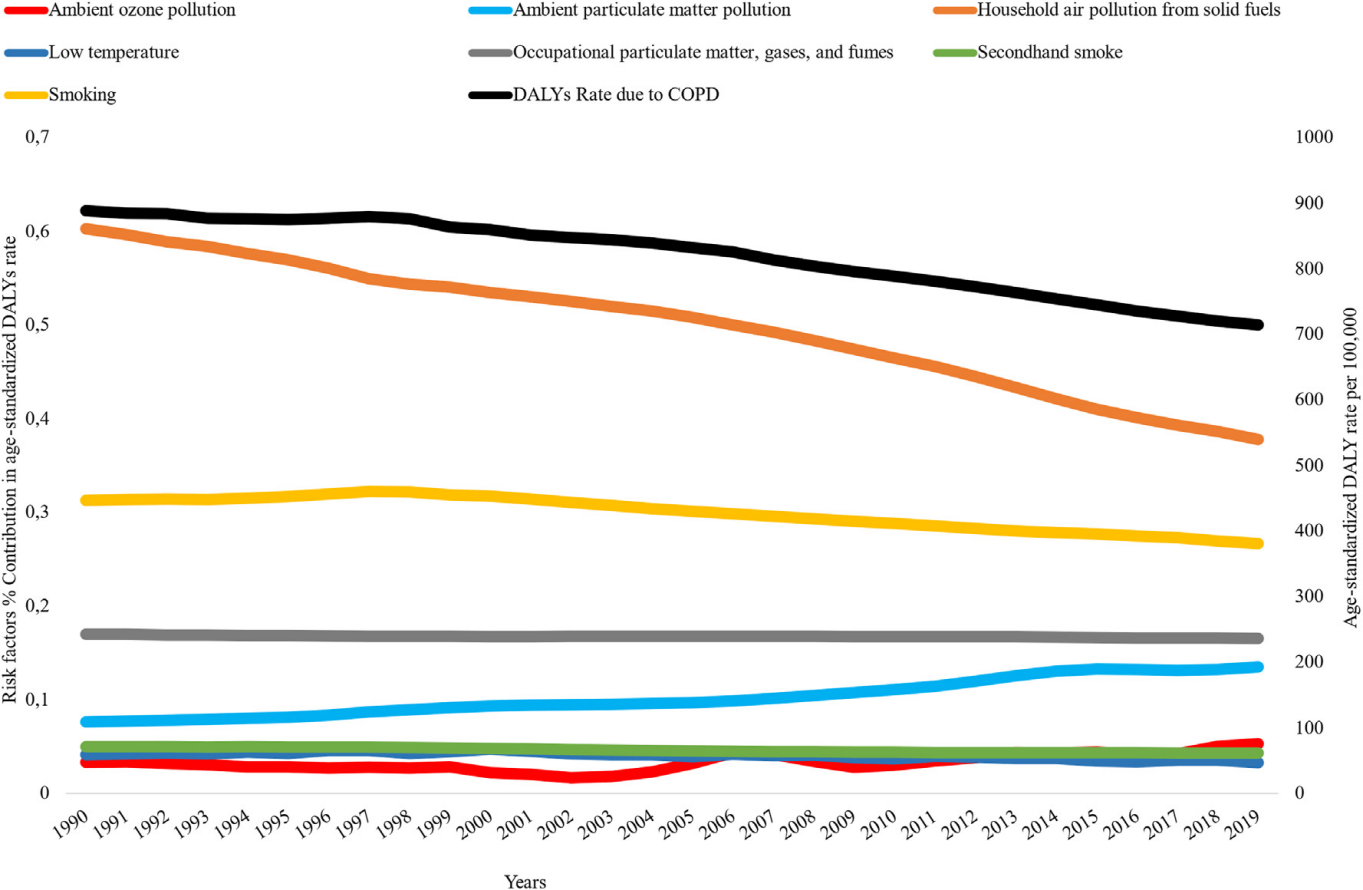
Trend in age-standardized DALYs rate due to COPD and risk factors percentage contribution in age-standardized DALYs rate in Sub-Saharan Africa, 1990–2019.

COPD related to household air pollution risk contributed to the highest rate of DALYs per 100,000 in Sub-Saharan Africa followed by smoking, occupational and ambient particulate matter pollution. In 2019, smoking and ambient particulate matter were ranked 1st and 2nd contributors for DALYs rate attributable to COPD across Southern Sub-Saharan Africa [Fig. 9]. The reduction of percentage contribution of household air pollution exhibited similar trend to the reduction of age-standardized DALY rate due to COPD in SubSaharan Africa (Fig. 10). In Somalia, household air pollution from solid fuel was responsible for 80–85% of age-standardized DALYs rate due to COPD (Appendix Table A7).

## Discussion

This GBD data analysis shows that the burden of COPD in SSA is substantial, with an estimated 10.3 million people having COPD (1.74% prevalence). The number of all-age prevalence cases of COPD in SSA increased by 117% in 2019 compared to 1990, whereas the age-standardized COPD prevalence rate showed a decline of 3.3%. There was a significant variation in COPD prevalence between regions and countries, with the highest age-standardized COPD prevalence rate was observed in Southern SSA, and in Sao Tome and Principe. COPD gave a significant contribution to the disease burden and about two-thirds of the premature mortality was due to COPD. In 2019, COPD was responsible for 715 DALY rate per 100,000 people across SSA, with the highest DALY observed in Central SSA.

Over the past 30 years, the age-standardized DALY rate due to COPD declined by 19.6% in SSA with the largest reduction observed in the Eastern SSA, and in Ethiopia. Household air pollution from solid fuel was the primary risk factors responsible for 270.4 DALY rate per 100,000 people due to COPD. There were large variations on risk factor contribution to the burden attributed to COPD by sex, region, and country. Amongst women, over half of deaths due to COPD in Eastern and Central SSA were attributed to household air pollution from solid fuel, whilst smoking contributed to 12.8% and 6% in each of these two areas. In Southern SSA, 28.2% of deaths due to COPD in women was explained by smoking, whilst in men, smoking was responsible for more than half of premature mortality and DALYs due to COPD.

As discussed by Adeloye D et al., aging alone can contribute as much as 31% to the increase of the prevalence of COPD in Africa.^7^ This could be one potential reason for the prevalence rate difference among all-age and age-standardized prevalence, the latter being adjusted for the age structure difference. Similar prevalence rate difference due to the difference in age structure and/or population growth had also been reported in other study.^32^ The prevalence of COPD reported in this GBD paper differs from the results from several population-based studies conducted in other countries from SSA.^7^^,^^12^^,^^14^, ^15^, ^16^, ^17^^,^^19^^,^^20^ Population-based estimates from the Burden of Obstructive Lung Disease (BOLD) study, using standardized protocols, reported a prevalence of COPD of 3.6% in Malawi^33^ and 7.7% in Nigeria.^34^ The estimates from this GBD report showed a slower prevalence of 1.54% and 1.38% in Malawi and Nigeria, respectively. The systematic analysis for GBD 2019 round was based on GBD 2016 iterations^3^ and in this study, COPD prevalence was estimated based on five distinct datapoints. The estimate in this GBD study is also based on country-level covariates such as HAQi and SEV for COPD which could contribute to explain the differences observed with the results of the BOLD study. These differences highlight the need to harmonize the methodologies used in the GBD framework to improve their representativeness in the general population.

Our study found that there was a significant variation in COPD prevalence between regions, and countries. Aaron et al. showed that known risk factors such as sex, age, and smoking explained 64% of variability in COPD prevalence.^35^ In our study, the highest age-standardized COPD prevalence was observed in Southern SSA. This might be partly driven by the 4–10 percentage increase observed in tobacco use in several countries in the region, including Botswana, Lesotho and Zimbabwe.^24^ Our results show an increasing COPD prevalence in those countries since 2017. Similarly, there was an increasing trend in COPD attributable to smoking in Sao Tome and Principe where the highest age-standardized COPD prevalence was observed compared to other Sub-Saharan countries. Tobacco was listed among the top 10 risks factors contributing to total number of disease burden in Sao Tome and Principe with an increase of 30% in 2019 from 2009.^24^ From 2009 to 2019, there was an increasing trend in tobacco attributable burden among countries in Central SSA with a percentage change increase from 6.4 to 36.6%.^24^ This increase in smoking might have contributed to the 12% increase in age-standardized prevalence rate of COPD observed in Central SSA between 1990 and 2019.

A recent study estimated that in low and middle income countries, as much as 8.6 million deaths from 61 conditions could be averted by a Universal Health Coverage (UHC) system.^36^ In SSA, countries differ in their coverage and effectiveness of UHC: Cape Verde, for example, delivers effective, essential health services with 62.2% performance on UHC effective coverage index,^37^ whilst Lesotho has a much lower performance of 38.7%. The differences in the performance of delivering essential health care services could also contribute to the variations in those countries having the lowest (Cape Verde) and highest (Lesotho) death rates due to COPD. The death rates have decreased over time; however, COPD cases have increased over time posing a heavy burden on Universal Health Coverage (UHC) systems. This could have a significant impact on SSA countries with weak health systems and has policy implications.

In a study conducted by Hystad et al., the use of solid fuel use was associated to fatal or nonfatal respiratory diseases compared with electricity or gas with a fully adjusted hazard ratios of 1.14.^38^ Another study also showed that COPD prevalence was higher among women using biomass, women aged >40 years, and was determined by years of exposure.^13^ In this GBD analysis, COPD attributable DALYs rate in most countries in Eastern SSA was mainly due to household air pollution from solid fuel. The highest reduction in DALY rate due to COPD attributed to household air pollution from solid fuel could be the potential reason of the difference in the reduction between Ethiopia and Ghana, where the latter had a stable trend over the past 30 years with a lowest DALY rate reduction.

In a recent systematic review, the use of improved cookstove (cleaner fuels) was associated with a lower risk of chronic respiratory symptoms, and with lower odds of COPD among women. The authors, however, acknowledged methodological limitations in the original studies included in the meta-analyses, such as measurement of air pollution, and study designs reduction.^39^^,^^40^ These limitations in the original surveys reduce the strength of the evidence, and their interpretation. Given the limitations of the GBD methodology, it is important to observe that recent population-based surveys from the BOLD study, collecting high quality spirometry to define COPD, found no evidence of an association between airflow obstruction and use of solid fuels for cooking or heating.^41^ These results are further supported by a recent prospective survey showing that exposure to air pollution was not associated with lung function decline. These findings fill an important scientific gap, as both lung function and air pollution were objectively measured, using standardized methods.^42^

The definition used for indoor biomass use in the GBD was drawn from a systematic review that reported publication bias in its studies, and from pooled data that showed 85% heterogeneity, therefore the findings on this risk factor need to be interpreted with caution. The potential difference between GBD estimates and findings from BOLD could also be explained by issues related to difference in study methodology, including variations in diagnostic criteria, the use of different spirometric cut-off measures, study settings, and data sources.

Our study provides up-to-date evidence on the burden of COPD across Sub-Saharan countries and regions, and the contribution of the major risk factors to the premature morbidity and mortality. Observing the increasing trends in COPD change over the past thirty years is important to improve the understanding of the status and progress being made in Africa, a region facing some of the greatest public health and climate change challenges. The subsequent implication is to identify and prioritize policy options using the evidence we have on the contribution of risk factors towards the achievements of sustainable development goals (SDG), particularly SDG 3.4.^1^ Our study has also show that the heterogeneous distribution of COPD and its inconsistencies highlighted the importance for further studies with standard definitions of airflow obstruction and of risk factors representative of the general population in SSA.

This analysis has some limitations, including the paucity of population-based studies where the prevalence estimate was derived using five datapoints and country-level covariates, which have been collected in other regional analyses.^32^ Albeit the GBD methodology has greatly contributed to improve our understanding of the distribution of disease and their impact on mortality, there are some methodological limitations that need to be considered. The GBD rely heavily on the WHO mortality data, but it is unlikely that these represent mortality associated with chronic airflow obstruction. Findings from systematic reviews are hindered by the limitations of the original studies, outcome definitions, and publication bias. The large heterogeneity between studies raises concerns about the suitability of the overall estimates from meta-analyses and about their usefulness. We were unable to examine the role of other important environmental risk factors such as diet, poverty, education, and history of tuberculosis, which have been shown to be important attributable factors for COPD in studies of the general population.^43^

In conclusion, this GBD analysis shows that the prevalence and mortality for COPD in SSA are a major public health challenge, and they give a significant contribution to premature morbidity and mortality across the region. Amongst the risk factors studied, household air pollution from solid fuel and smoking were the major contributors to COPD related burden. The heterogenous distribution of COPD across countries in the region suggest further research to figure out reasons for the heterogeneity including other important environmental and socio-demographic factors. In addition, the limitations from the GBD methodology need to be taken into consideration, as increasing population-based evidence fails to confirm an association between household pollution and COPD, therefore the findings from the GBD need to be interpreted with caution. The findings of this study can inform global health stakeholders and policymakers about the public health implications of COPD in the population of Sub-Saharan Africa. As a result, a more comprehensive study on a wide range of causes of COPD that may be unique to different parts of the world could be incorporated into this type of research.

## Contributors

All authors in the writing team (Mulubirhan Assefa Alemayohu, Maria Elisabetta Zanolin, Lucia Cazzoletti, Peter Nyasulu, and Vanessa Garcia-Larsen) contributed to the writing of all versions of the manuscript and approved the final version. MAA conceived and designed the article, accessed, and verified the data, prepared the original draft, and reviewed Collaborators’ comments. MEZ, LC, PN, and VGL assisted with the design, critically reviewed, and contributed to the manuscript. MEZ, and LC also accessed and verified the data. Please see Appendix B for more detailed information about individual author contributions to the research, divided into the following categories: providing data or critical feedback on data sources; developing methods or computational machinery; providing critical feedback on methods or results; drafting the manuscript or revising it critically for important intellectual content; and managing the estimation or publications process.

## Data sharing statement

The data used for this analysis is publicly available https://ghdx.healthdata.org/gbd-2019 and can be downloaded and used according to the Global Health Data Exchange policy.

## Declaration of interests

All authors in the writing team declare no competing interests.

## Appendix A Supplementary data

Supplementary Tables

PubMed SSA COPD
