# Supplementary material for: Burden and risk factors of chronic obstructive pulmonary disease in Sub-Saharan African countries, 1990–2019: a systematic analysis for the Global Burden of disease study 2019
Source: eClinicalMedicine. 2023 Oct 2;64:102215. doi: 10.1016/j.eclinm.2023.102215 (PMC10550520; doi:10.1016/j.eclinm.2023.102215)
Supplement: Appendix A [file mmc1.docx]

**Burden and risk factors of chronic obstructive pulmonary disease in Sub-Saharan African countries, 1990-2019: A systematic analysis for the Global Burden of Disease Study 2019**

Mulubirhan Assefa Alemayohu, PhD^1,2,3^ Maria Elisabetta Zanolin, PhD^1^ Lucia Cazzoletti, PhD^1^ Peter Nyasulu, PhD^4^ Vanessa Garcia-Larsen, PhD^5^ and ***GBD 2019 Sub-Saharan COPD Collaborators*^*^**

^1^ Unit of Epidemiology and Medical Statistics, University of Verona, Italy

^2^ School Public Health, Mekelle University, Ethiopia

^3^ Biostatistics and Clinical Epidemiology Unit, Department of Public Health, Experimental and Forensic Medicin, University of Pavia, 27100 Pavia, Italy

^4^ Faculty of Medicine and Health Sciences, Stellenbosch University, Cape Town, South Africa

^5^ Department of International Health, The Johns Hopkins Bloomberg School of Public Health, Baltimore, US

^*^ Authors list provided at the end of the manuscript.

###### **Supplementary Files**

**Appendix Table A.1** Age Standardized prevalence of COPD and YLD in 2019 and percentage change in age-standardized prevalence and YLDs by location and sex.

|  | Age-standardized Prevalence Rate per 100,000 in 2019 (95%UI) | | | Percentage Change in age standardized Prevalence rate, 1990–2019 | | | Age-standardized YLDs Rate in 2019 (95%UI) | | | Percentage Change in age standardized YLDs rate, 1990–2019 | | |
| --- | --- | --- | --- | --- | --- | --- | --- | --- | --- | --- | --- | --- |
|  | Both | Female | Male | Both | Female | Male | Both | Female | Male | Both | Female | Male |
| Sub-Saharan Africa | 1705  (1599 to 1820) | 1656  (1550 to 1771) | 1769  (1665 to 1879) | -3.3 | -5.0 | -1.8 | 205  (171 to 231) | 213  (178 to 245) | 196  (162 to 225) | -3.3 | -4.9 | -2.0 |
| Central Sub-Saharan Africa | 1943  (1817 to 2076) | 2009  (1866 to 2162) | 1841  (1720 to 1968) | 12.2 | 11.9 | 11.1 | 235  (197 to 269) | 261  (215 to 305) | 202  (167 to 233) | 13.0 | 12.5 | 11.6 |
| Eastern Sub-Saharan Africa | 1503  (1408 to 1609) | 1411  (1320 to 1513) | 1616  (1509 to 1728) | -4.7 | -6.6 | -2.5 | 183  (153 to 209) | 186  (155 to 216) | 182  (152 to 208) | -4.2 | -6.1 | -1.6 |
| Southern Sub-Saharan Africa | 2244  (2090 to 2412) | 1919  (1773 to 2076) | 2771  (2580 to 2980) | -9.7 | -11.5 | -6.2 | 288  (239 to 329) | 282  (234 to 329) | 306  (253 to 353) | -10.6 | -12.4 | -7.0 |
| Western Sub-Saharan Africa | 1618  (1522 to 1721) | 1665  (1559 to 1779) | 1571  (1481 to 1665) | -4.5 | -6.8 | -3.3 | 191  (161 to 216) | 202  (169 to 233) | 179  (149 to 205) | -4.0 | -6.5 | -2.7 |
| Angola | 1758  (1645 to 1871) | 1834  (1702 to 1975) | 1656  (1549 to 1771) | 1.9 | -0.6 | 3.9 | 215  (177 to 244) | 240  (197 to 282) | 183  (149 to 213) | 2.9 | -0.4 | 4.0 |
| Benin | 1928  (1809 to 2049) | 2003  (1868 to 2133) | 1856  (1749 to 1974) | 1.3 | 7.2 | -4.9 | 229  (192 to 261) | 246  (200 to 286) | 211  (172 to 245) | 1.8 | 7.4 | -4.1 |
| Botswana | 2266  (2127 to 2409) | 2113  (1965 to 2254) | 2634  (2474 to 2818) | 0.0 | 21.0 | -12.6 | 291  (244 to 332) | 306  (254 to 358) | 290  (241 to 335) | 0.7 | 18.6 | -14.2 |
| Burkina Faso | 1452  (1372 to 1534) | 1491  (1402 to 1580) | 1412  (1327 to 1502) | 8.0 | 4.1 | 13.2 | 174  (146 to 198) | 185  (151 to 216) | 162  (134 to 187) | 8.8 | 4.5 | 14.9 |
| Burundi | 1758  (1653 to 1871) | 1609  (1507 to 1718) | 1917  (1797 to 2042) | -8.2 | -5.7 | -12.1 | 212  (177 to 242) | 212  (174 to 247) | 214  (176 to 249) | -8.6 | -5.4 | -11.9 |
| Côte d'Ivoire | 1698  (1601 to 1805) | 1680  (1576 to 1795) | 1728  (1626 to 1836) | -4.5 | -2.6 | -5.9 | 201  (168 to 228) | 207  (171 to 241) | 196  (162 to 226) | -2.9 | -1.4 | -4.4 |
| Cape Verde | 1514  (1437 to 1598) | 1425  (1330 to 1518) | 1614  (1525 to 1707) | -11.6 | -5.4 | -19.7 | 186  (155 to 210) | 193  (158 to 224) | 177  (144 to 205) | -12.3 | -6.3 | -19.9 |
| Cameroon | 2216  (2082 to 2360) | 2283  (2129 to 2444) | 2155  (2024 to 2291) | -1.7 | -6.2 | 3.9 | 260  (219 to 297) | 278  (230 to 326) | 242  (199 to 281) | -1.5 | -6.1 | 4.3 |
| Central African Republic | 1918  (1786 to 2054) | 1846  (1700 to 1996) | 1973  (1837 to 2110) | -0.1 | -1.1 | 0.5 | 231  (192 to 264) | 240  (200 to 280) | 216  (177 to 253) | 0.4 | -0.8 | 0.9 |
| Chad | 1680  (1580 to 1784) | 1732  (1616 to 1853) | 1646  (1548 to 1749) | 2.4 | 5.3 | -0.2 | 198  (166 to 225) | 212  (174 to 247) | 186  (154 to 216) | 1.5 | 4.4 | -0.5 |
| Comoros | 1475  (1379 to 1569) | 1364  (1274 to 1467) | 1621  (1509 to 1728) | -3.0 | -2.7 | -2.1 | 181  (151 to 207) | 181  (151 to 211) | 183  (150 to 213) | -2.7 | -2.7 | -2.1 |

| *Appendix Table A.1 … Continued* | | | | | | | | | | | | |
| --- | --- | --- | --- | --- | --- | --- | --- | --- | --- | --- | --- | --- |
|  | Age-standardized Prevalence Rate per 100,000 in 2019 (95%UI) | | | Percentage Change in age standardized Prevalence rate, 1990–2019 | | | Age-standardized YLDs Rate in 2019 (95%UI) | | | Percentage Change in age standardized YLDs rate, 1990–2019 | | |
|  | Both | Female | Male | Both | Female | Male | Both | Female | Male | Both | Female | Male |
| Congo | 2069  (1936 to 2209) | 2430  (2259 to 2603) | 1671  (1555 to 1799) | 1.1 | 11.2 | -9.9 | 253  (209 to 289) | 315  (260 to 369) | 184  (149 to 214) | 1.2 | 10.9 | -9.8 |
| Democratic Republic of the Congo | 1999  (1866 to 2146) | 2052  (1896 to 2220) | 1906  (1771 to 2049) | 17.5 | 16.3 | 16.7 | 241  (202 to 276) | 266  (219 to 312) | 209  (172 to 243) | 18.1 | 17.2 | 17.4 |
| Equatorial Guinea | 1850  (1711 to 1992) | 2067  (1898 to 2253) | 1546  (1428 to 1663) | 8.7 | 21.4 | -8.7 | 225  (188 to 258) | 267  (220 to 313) | 169  (138 to 197) | 10.3 | 21.9 | -8.2 |
| Eritrea | 1814  (1697 to 1940) | 1551  (1447 to 1669) | 2180  (2025 to 2342) | 2.0 | 4.8 | -2.0 | 219  (181 to 251) | 204  (168 to 239) | 242  (196 to 283) | 2.3 | 4.6 | -1.6 |
| Eswatini | 2477  (2348 to 2606) | 1976  (1837 to 2122) | 3333  (3163 to 3491) | -4.8 | -8.3 | 0.8 | 314  (262 to 355) | 287  (237 to 336) | 368  (306 to 422) | -6.5 | -9.7 | -0.8 |
| Ethiopia | 1182  (1068 to 1303) | 1136  (1030 to 1252) | 1225  (1099 to 1361) | -27.5 | -25.3 | -30.6 | 145  (119 to 168) | 151  (123 to 178) | 139  (115 to 162) | -26.0 | -24.1 | -28.7 |
| Gabon | 1637  (1530 to 1752) | 1478  (1372 to 1599) | 1828  (1706 to 1953) | -0.6 | -6.6 | 5.8 | 197  (164 to 224) | 193  (159 to 225) | 201  (164 to 234) | -1.0 | -6.3 | 5.8 |
| Gambia | 2004  (1883 to 2133) | 2119  (1976 to 2266) | 1902  (1786 to 2022) | 5.5 | 8.9 | 1.0 | 237  (197 to 271) | 259  (213 to 301) | 217  (177 to 251) | 5.3 | 8.4 | 0.9 |
| Ghana | 1734  (1638 to 1833) | 1607  (1509 to 1704) | 1914  (1799 to 2025) | 23.1 | 5.6 | 47.8 | 199  (165 to 224) | 178  (147 to 206) | 226  (186 to 260) | 23.6 | 5.3 | 46.8 |
| Guinea | 1979  (1859 to 2108) | 1981  (1844 to 2120) | 1989  (1865 to 2117) | 14.3 | 7.9 | 21.8 | 236  (197 to 271) | 245  (202 to 287) | 228  (187 to 267) | 14.0 | 7.9 | 21.3 |
| Guinea-Bissau | 2032  (1908 to 2165) | 1992  (1865 to 2132) | 2092  (1956 to 2234) | -0.4 | 5.3 | -5.9 | 241  (200 to 275) | 244  (203 to 284) | 237  (192 to 277) | 0.4 | 5.2 | -5.2 |
| Kenya | 1730  (1590 to 1893) | 1651  (1521 to 1800) | 1845  (1693 to 2017) | 0.7 | -7.6 | 11.9 | 211  (174 to 243) | 217  (180 to 253) | 206  (169 to 239) | 0.5 | -7.3 | 10.8 |
| Lesotho | 2815  (2671 to 2961) | 2500  (2329 to 2670) | 3369  (3204 to 3534) | 11.2 | 20.8 | 3.2 | 361  (301 to 410) | 361  (298 to 422) | 374  (307 to 428) | 10.7 | 18.0 | 1.6 |
| Liberia | 1520  (1431 to 1609) | 1750  (1639 to 1864) | 1306  (1212 to 1404) | 18.5 | 20.9 | 14.8 | 179  (150 to 203) | 212  (175 to 247) | 147  (121 to 171) | 18.5 | 20.5 | 14.0 |
| Madagascar | 2171  (2027 to 2319) | 2121  (1968 to 2278) | 2235  (2086 to 2386) | 11.2 | 7.1 | 16.2 | 265  (218 to 302) | 279  (229 to 327) | 251  (203 to 293) | 11.3 | 7.3 | 16.7 |
| Malawi | 1493  (1405 to 1587) | 1262  (1171 to 1353) | 1818  (1703 to 1933) | 5.7 | -4.6 | 19.6 | 181  (151 to 207) | 167  (138 to 195) | 204  (168 to 236) | 5.2 | -4.0 | 19.3 |
| Mali | 2327  (2176 to 2480) | 2439  (2263 to 2615) | 2224  (2077 to 2376) | 9.7 | 11.2 | 8.3 | 275  (232 to 317) | 299  (246 to 351) | 253  (207 to 296) | 10.0 | 11.6 | 9.1 |
| Mauritania | 1715  (1616 to 1824) | 1928  (1806 to 2057) | 1500  (1398 to 1605) | -6.1 | -2.8 | -10.0 | 205  (173 to 233) | 238  (197 to 277) | 172  (142 to 199) | -6.0 | -2.5 | -9.5 |
| Mauritius | 1985  (1882 to 2086) | 1400  (1310 to 1492) | 2717  (2563 to 2876) | -12.1 | -17.3 | -9.9 | 202  (164 to 232) | 143  (116 to 168) | 274  (220 to 320) | -12.6 | -17.8 | -10.7 |
| Mozambique | 1558  (1460 to 1651) | 1269  (1180 to 1363) | 1937  (1816 to 2054) | 25.0 | 17.7 | 34.1 | 187  (156 to 214) | 167  (139 to 195) | 215  (177 to 248) | 23.8 | 17.6 | 33.5 |
| Namibia | 2191  (2063 to 2330) | 1670  (1543 to 1800) | 2982  (2812 to 3164) | -7.5 | -14.3 | 2.0 | 278  (232 to 316) | 245  (201 to 286) | 332  (275 to 385) | -8.3 | -14.6 | 1.2 |
| Niger | 1719  (1607 to 1832) | 1767  (1644 to 1895) | 1670  (1565 to 1776) | 5.2 | 5.3 | 3.1 | 205  (171 to 233) | 218  (179 to 254) | 191  (157 to 223) | 5.7 | 5.3 | 3.2 |
| Nigeria | 1356  (1245 to 1478) | 1456  (1334 to 1596) | 1241  (1134 to 1344) | -17.6 | -17.6 | -20.5 | 161  (134 to 185) | 178  (146 to 209) | 142  (116 to 164) | -16.6 | -16.8 | -19.3 |
| Rwanda | 1806  (1693 to 1938) | 1680  (1565 to 1810) | 2018  (1892 to 2161) | -5.3 | -2.5 | -6.7 | 220  (183 to 252) | 221  (184 to 259) | 225  (184 to 261) | -4.8 | -2.2 | -6.3 |
| Sao Tome and Principe | 3392  (3222 to 3560) | 3202  (3031 to 3388) | 3626  (3437 to 3801) | 33.8 | 26.4 | 39.7 | 395  (329 to 444) | 389  (321 to 450) | 404  (333 to 467) | 32.1 | 25.1 | 38.4 |
| Senegal | 1759  (1655 to 1861) | 1686  (1581 to 1798) | 1845  (1729 to 1951) | 1.6 | 4.1 | -0.6 | 207  (173 to 235) | 207  (169 to 240) | 208  (170 to 241) | 2.0 | 4.0 | -0.5 |
| Seychelles | 2171  (2057 to 2291) | 1557  (1455 to 1658) | 2887  (2725 to 3064) | 15.8 | 12.2 | 15.1 | 222  (181 to 255) | 160  (130 to 188) | 293  (236 to 342) | 14.4 | 10.3 | 13.1 |
| Sierra Leone | 1875  (1767 to 1987) | 2024  (1899 to 2155) | 1737  (1638 to 1845) | 12.1 | 22.7 | 2.1 | 222  (185 to 251) | 249  (204 to 289) | 197  (162 to 228) | 12.1 | 22.1 | 2.1 |
| Somalia | 1518  (1409 to 1628) | 1418  (1311 to 1537) | 1686  (1565 to 1804) | -4.3 | -1.7 | -4.5 | 186  (153 to 214) | 187  (155 to 219) | 189  (155 to 221) | -3.6 | -1.1 | -5.0 |
| South Africa | 2256  (2088 to 2440) | 1920  (1766 to 2089) | 2803  (2591 to 3038) | -12.7 | -15.5 | -8.5 | 289  (240 to 331) | 283  (233 to 333) | 309  (255 to 359) | -13.7 | -16.0 | -9.1 |
| South Sudan | 1568  (1458 to 1684) | 1471  (1362 to 1590) | 1655  (1537 to 1775) | -3.9 | -4.7 | -3.8 | 187  (157 to 214) | 190  (159 to 223) | 184  (152 to 213) | -3.1 | -4.5 | -3.2 |
| Sudan | 1863  (1748 to 1989) | 1692  (1584 to 1812) | 2003  (1872 to 2147) | 18.4 | 9.9 | 24.9 | 172  (139 to 200) | 168  (136 to 196) | 175  (139 to 208) | 17.0 | 9.8 | 24.1 |
| Togo | 1969  (1850 to 2095) | 1853  (1736 to 1976) | 2138  (1995 to 2274) | 0.8 | -3.8 | 7.0 | 234  (196 to 266) | 229  (188 to 266) | 242  (200 to 283) | 1.3 | -3.4 | 7.1 |
| Uganda | 1556  (1450 to 1662) | 1396  (1287 to 1510) | 1789  (1667 to 1908) | -0.4 | -11.0 | 14.2 | 190  (157 to 216) | 184  (154 to 215) | 200  (163 to 233) | 0.5 | -9.8 | 14.9 |
| United Republic of Tanzania | 1299  (1208 to 1385) | 1289  (1202 to 1377) | 1321  (1221 to 1415) | 5.4 | 4.3 | 6.5 | 160  (133 to 184) | 171  (139 to 200) | 150  (123 to 175) | 6.0 | 5.6 | 7.1 |
| Zambia | 1650  (1551 to 1755) | 1514  (1418 to 1617) | 1816  (1701 to 1936) | 8.4 | 0.9 | 17.8 | 200  (167 to 227) | 199  (164 to 232) | 203  (165 to 235) | 8.1 | 0.5 | 17.3 |
| Zimbabwe | 1992  (1861 to 2134) | 1779  (1642 to 1924) | 2299  (2141 to 2454) | 2.4 | 4.5 | 2.7 | 260  (214 to 296) | 262  (213 to 306) | 260  (213 to 301) | 3.2 | 4.0 | 2.4 |

***Notes****: YLDs - years lived with disabilities. 95% UI - 95% uncertainty intervals. COPD - chronic obstructive pulmonary disease*

**Appendix Table A.2** Age Standardized Death and YLL rate in 2019 and percentage change in age-standardized Death and YLLs by location and sex

|  | Age-standardized Death rate in 2019, (95%UI) | | | Percentage Change in Age-standardized Death rate, 1990–2019 | | | Age-standardized YLLs rate in 2019, (95%UI) | | | Percentage Change in Age-standardized YLLs rate, 1990–2019 | | |
| --- | --- | --- | --- | --- | --- | --- | --- | --- | --- | --- | --- | --- |
|  | Both | Female | Male | Both | Female | Male | Both | Female | Male | Both | Female | Male |
| Sub-Saharan Africa - WB | 29  (25 to 33) | 21  (15 to 27) | 40  (33 to 45) | -21.6 | -16.0 | -21.6 | 510  (436 to 587) | 364  (259 to 474) | 684  (565 to 801) | -24.7 | -23.8 | -24.2 |
| Central Sub-Saharan Africa | 43  (30 to 67) | 38  (19 to 75) | 49  (33 to 65) | -17.3 | -11.6 | -22.2 | 729  (509 to 1078) | 628  (319 to 1198) | 859  (550 to 1161) | -21.9 | -16.2 | -24.9 |
| Eastern Sub-Saharan Africa | 29  (25 to 34) | 20  (14 to 26) | 42  (34 to 50) | -27.5 | -23.1 | -26.3 | 524  (444 to 617) | 353  (249 to 476) | 725  (582 to 890) | -31.4 | -29.8 | -30.4 |
| Southern Sub-Saharan Africa | 32  (29 to 35) | 22  (19 to 26) | 50  (45 to 54) | -17.9 | -18.5 | -13.8 | 550  (503 to 603) | 358  (295 to 417) | 842  (771 to 918) | -19.1 | -21.0 | -15.8 |
| Western Sub-Saharan Africa | 24  (21 to 28) | 16  (12 to 20) | 33  (27 to 40) | -17.2 | -23.8 | -19.5 | 428  (355 to 496) | 300  (220 to 370) | 569  (450 to 691) | -20.4 | -24.2 | -18.6 |
| Angola | 31  (23 to 39) | 27  (15 to 41) | 35  (27 to 43) | -35.4 | -34.1 | -36.4 | 515  (384 to 654) | 449  (238 to 648) | 598  (466 to 759) | -41.8 | -40.1 | -41.7 |
| Benin | 26  (20 to 33) | 18  (13 to 25) | 37  (27 to 48) | -31.6 | -28.0 | -28.8 | 464  (352 to 612) | 334  (229 to 472) | 628  (452 to 839) | -32.3 | -29.8 | -31.4 |
| Botswana | 39  (27 to 52) | 25  (13 to 36) | 64  (46 to 86) | -30.4 | -28.6 | -28.1 | 706  (477 to 947) | 427  (214 to 625) | 1125  (800 to 1547) | -30.5 | -29.4 | -29.1 |
| Burkina Faso | 19  (16 to 23) | 13  (10 to 18) | 27  (21 to 34) | -17.4 | -18.8 | -10.0 | 350  (282 to 434) | 262  (180 to 345) | 466  (357 to 606) | -14.8 | -17.1 | -10.0 |
| Burundi | 43  (32 to 55) | 29  (19 to 42) | 58  (41 to 78) | -28.3 | -25.6 | -34.1 | 784  (588 to 1033) | 542  (336 to 815) | 1025 (710 to 1455) | -34.0 | -31.0 | -39.1 |
| Côte d'Ivoire | 25  (20 to 31) | 16  (11 to 21) | 36  (26 to 45) | -34.2 | -27.3 | -33.3 | 447  (335 to 574) | 283  (192 to 393) | 612  (440 to 793) | -34.7 | -30.3 | -35.2 |
| Cape Verde | 18  (14 to 25) | 13  (9 to 19) | 25  (21 to 34) | -48.6 | -48.0 | -47.9 | 296  (243 to 403) | 205  (144 to 303) | 422  (352 to 581) | -53.6 | -55.0 | -52.8 |
| Cameroon | 25  (18 to 32) | 17  (11 to 24) | 35  (23 to 46) | -34.2 | -34.6 | -31.4 | 453  (307 to 599) | 314  (196 to 441) | 616  (379 to 824) | -32.9 | -36.0 | -29.9 |
| Central African Republic | 53  (35 to 79) | 42  (22 to 81) | 68  (44 to 92) | -14.5 | -10.6 | -16.0 | 1003  (669 to 1424) | 748  (371 to 1361) | 1334  (846 to 1818) | -15.7 | -12.2 | -16.6 |
| Chad | 31  (23 to 40) | 22  (14 to 30) | 40  (27 to 54) | -13.9 | -15.4 | -16.7 | 568  (417 to 741) | 413  (271 to 590) | 706  (476 to 985) | -15.4 | -18.4 | -17.3 |

| *Appendix Table A.2 … Continued* | | | | | | | | | | | | |
| --- | --- | --- | --- | --- | --- | --- | --- | --- | --- | --- | --- | --- |
|  | Age-standardized Deaths rate in 2019, (95%UI) | | | Percentage Change in Age-standardized Deaths rate, 1990–2019 | | | Age-standardized YLLs rate in 2019, (95%UI) | | | Percentage Change in Age-standardized YLLs rate, 1990–2019 | | |
|  | Both | Female | Male | Both | Female | Male | Both | Female | Male | Both | Female | Male |
| Comoros | 26  (20 to 33) | 17  (11 to 26) | 38  (29 to 48) | -33.3 | -26.1 | -35.6 | 451  (347 to 588) | 313  (195 to 484) | 626  (474 to 811) | -35.1 | -25.5 | -37.4 |
| Congo | 35  (26 to 44) | 30  (15 to 47) | 41  (21 to 51) | -35.2 | -30.2 | -42.3 | 579  (420 to 748) | 488  (248 to 722) | 684  (349 to 885) | -41.7 | -34.3 | -48.3 |
| Democratic Republic of the Congo | 47  (30 to 77) | 42  (21 to 88) | 54  (31 to 74) | -11.3 | -4.5 | -15.6 | 801  (520 to 1251) | 696  (341 to 1412) | 938  (527 to 1320) | -14.3 | -8.1 | -17.1 |
| Equatorial Guinea | 30  (20 to 51) | 25  (12 to 64) | 38  (20 to 52) | -42.3 | -34.2 | -47.9 | 474  (303 to 771) | 380  (179 to 930) | 611  (314 to 845) | -52.1 | -44.4 | -56.3 |
| Eritrea | 33  (23 to 42) | 24  (12 to 36) | 49  (32 to 65) | -19.5 | -11.1 | -29.0 | 621  (435 to 799) | 452  (211 to 677) | 893  (585 to 1204) | -26.5 | -16.5 | -33.9 |
| Eswatini | 42  (31 to 55) | 25  (14 to 37) | 78  (62 to 95) | -27.6 | -28.6 | -22.0 | 779  (572 to 1026) | 403  (216 to 621) | 1420  (1110 to 1797) | -27.1 | -32.7 | -20.0 |
| Ethiopia | 28  (23 to 33) | 19  (13 to 25) | 37  (27 to 47) | -39.1 | -36.7 | -41.3 | 484  (386 to 573) | 338  (231 to 447) | 623  (452 to 803) | -48.7 | -46.8 | -49.7 |
| Gabon | 26  (18 to 33) | 19  (9 to 27) | 35  (21 to 49) | -35.0 | -36.7 | -36.4 | 427  (295 to 560) | 295  (132 to 411) | 593  (348 to 844) | -39.7 | -40.4 | -40.3 |
| Gambia | 30  (23 to 38) | 22  (15 to 32) | 42  (31 to 54) | -11.8 | -4.3 | -14.3 | 542  (405 to 697) | 391  (257 to 584) | 717  (515 to 942) | -12.4 | -8.4 | -13.4 |
| Ghana | 26  (16 to 32) | 11  (8 to 14) | 48  (24 to 60) | -10.3 | -31.3 | 6.7 | 471  (281 to 592) | 203  (136 to 262) | 839  (438 to 1067) | -10.5 | -34.9 | 7.8 |
| Guinea | 31  (24 to 39) | 23  (16 to 32) | 40  (28 to 52) | -16.2 | -20.7 | -11.1 | 574  (432 to 733) | 439  (293 to 631) | 713  (498 to 944) | -14.6 | -24.0 | -7.3 |
| Guinea-Bissau | 34  (26 to 44) | 23  (16 to 32) | 51  (35 to 67) | -32.0 | -20.7 | -31.1 | 671  (491 to 872) | 446  (297 to 633) | 964  (637 to 1295) | -33.1 | -24.7 | -33.6 |
| Kenya | 31  (24 to 41) | 22  (14 to 37) | 44  (31 to 60) | -6.1 | -8.3 | 4.8 | 542  (422 to 713) | 381  (241 to 642) | 748  (521 to 1027) | -4.4 | -13.6 | 6.7 |
| Lesotho | 71  (50 to 95) | 52  (28 to 79) | 104  (73 to 140) | -4.1 | -1.9 | -8.0 | 1315  (921 to 1803) | 918  (467 to 1435) | 1956  (1353 to 2673) | -2.6 | -0.8 | -2.4 |
| Liberia | 18  (14 to 25) | 16  (11 to 24) | 21  (14 to 28) | -18.2 | -15.8 | -12.5 | 325  (236 to 453) | 301  (199 to 447) | 348  (220 to 495) | -19.2 | -20.6 | -17.3 |
| Madagascar | 43  (31 to 56) | 30  (19 to 45) | 59  (42 to 79) | -8.5 | 0.0 | -9.2 | 775  (557 to 1037) | 569  (354 to 854) | 1011  (711 to 1393) | -12.2 | -5.3 | -11.9 |
| Malawi | 25  (20 to 30) | 16  (10 to 21) | 40  (32 to 51) | -19.4 | -23.8 | -9.1 | 449  (352 to 550) | 275  (182 to 385) | 697  (537 to 912) | -21.4 | -30.6 | -10.8 |
| Mali | 34  (26 to 42) | 25  (14 to 36) | 42  (32 to 55) | -10.5 | -13.8 | -12.5 | 667  (482 to 866) | 512  (273 to 758) | 813  (593 to 1093) | -14.6 | -17.0 | -14.7 |
| Mauritania | 19  (15 to 23) | 15  (10 to 20) | 22  (17 to 28) | -38.7 | -34.8 | -45.0 | 308  (231 to 401) | 269  (179 to 368) | 346  (258 to 459) | -45.7 | -40.9 | -51.1 |
| Mauritius | 19  (15 to 25) | 11  (8 to 17) | 31  (25 to 38) | -38.7 | -35.3 | -40.4 | 298  (234 to 385) | 161  (116 to 260) | 479  (377 to 599) | -41.3 | -36.9 | -44.0 |
| Mozambique | 25  (20 to 33) | 14  (9 to 21) | 43  (33 to 60) | -7.4 | -12.5 | 7.5 | 471  (355 to 632) | 237  (147 to 357) | 792  (593 to 1111) | -2.9 | -17.1 | 10.2 |
| Namibia | 45  (34 to 58) | 27  (16 to 43) | 75  (59 to 89) | -28.6 | -27.0 | -25.0 | 777  (585 to 1008) | 430  (255 to 696) | 1289  (1012 to 1567) | -30.4 | -32.1 | -25.9 |
| Niger | 31  (22 to 43) | 24  (16 to 34) | 38  (25 to 61) | -18.4 | -11.1 | -25.5 | 547  (389 to 790) | 446  (283 to 645) | 655  (418 to 1087) | -23.6 | -17.1 | -27.4 |
| Nigeria | 22  (17 to 27) | 15  (10 to 19) | 29  (20 to 39) | -12.0 | -16.7 | -17.1 | 362  (274 to 456) | 267  (178 to 357) | 464  (319 to 640) | -19.0 | -21.0 | -20.0 |
| Rwanda | 36  (28 to 46) | 26  (17 to 42) | 53  (38 to 70) | -41.9 | -36.6 | -41.1 | 629  (490 to 816) | 458  (302 to 765) | 899  (643 to 1194) | -48.5 | -45.9 | -47.4 |
| Sao Tome and Principe | 58  (44 to 72) | 41  (22 to 57) | 79  (57 to 106) | 0.0 | -2.4 | -4.8 | 1014  (761 to 1261) | 720  (402 to 1009) | 1346  (952 to 1823) | 0.1 | -6.7 | 0.7 |
| Senegal | 26  (20 to 31) | 18  (13 to 23) | 35  (27 to 45) | -27.8 | -21.7 | -30.0 | 451  (350 to 567) | 322  (227 to 420) | 595  (445 to 794) | -29.8 | -25.1 | -30.7 |
| Seychelles | 25  (21 to 29) | 16  (12 to 20) | 38  (30 to 46) | -24.2 | -11.1 | -33.3 | 411  (335 to 479) | 241  (169 to 301) | 618  (487 to 749) | -28.3 | -18.6 | -35.8 |
| Sierra Leone | 27  (20 to 35) | 21  (14 to 30) | 35  (24 to 46) | -25.0 | -8.7 | -27.1 | 494  (359 to 658) | 396  (258 to 580) | 599  (404 to 842) | -23.2 | -9.8 | -28.6 |
| Somalia | 40  (26 to 63) | 27  (14 to 50) | 62  (39 to 119) | -23.1 | -10.0 | -22.5 | 771  (491 to 1219) | 517  (269 to 967) | 1161  (699 to 2241) | -24.5 | -15.9 | -22.9 |
| South Africa | 32  (29 to 36) | 22  (19 to 27) | 49  (45 to 54) | -15.8 | -15.4 | -14.0 | 536  (490 to 605) | 350  (304 to 437) | 819  (746 to 908) | -19.3 | -21.9 | -16.2 |
| South Sudan | 27  (18 to 36) | 13  (8 to 21) | 38  (26 to 54) | -25.0 | -31.6 | -29.6 | 456  (305 to 638) | 243  (141 to 403) | 644  (417 to 921) | -31.4 | -32.1 | -31.0 |
| Sudan | 29  (19 to 40) | 19  (12 to 31) | 37  (21 to 53) | -21.6 | -20.8 | -24.5 | 510  (332 to 725) | 331  (201 to 537) | 661  (369 to 956) | -27.4 | -30.2 | -27.6 |
| Togo | 26  (20 to 34) | 19  (13 to 27) | 40  (27 to 52) | -25.7 | -26.9 | -14.9 | 479  (350 to 633) | 339  (233 to 489) | 696  (466 to 957) | -25.0 | -32.2 | -13.8 |
| Uganda | 30  (22 to 39) | 18  (12 to 29) | 50  (33 to 65) | -31.8 | -28.0 | -25.4 | 537  (390 to 704) | 313  (201 to 500) | 856  (563 to 1137) | -32.5 | -29.0 | -28.0 |
| United Republic of Tanzania | 21  (17 to 25) | 15  (10 to 20) | 29  (23 to 37) | -19.2 | -16.7 | -21.6 | 381  (300 to 458) | 275  (187 to 381) | 506  (382 to 647) | -21.1 | -16.2 | -23.1 |
| Zambia | 29  (23 to 35) | 18  (12 to 25) | 43  (34 to 52) | -19.4 | -21.7 | -10.4 | 520  (407 to 638) | 329  (215 to 463) | 747  (571 to 930) | -21.1 | -26.2 | -11.6 |
| Zimbabwe | 24  (17 to 31) | 18  (8 to 26) | 34  (25 to 44) | -7.7 | -5.3 | -10.5 | 404  (279 to 525) | 276  (125 to 415) | 594  (406 to 780) | -7.8 | -6.1 | -3.4 |

*Notes: YLLs - years life lost. 95% UI - 95% uncertainty intervals. COPD - chronic obstructive pulmonary disease*

**Appendix Table A.3** Age Standardized DALY due to COPD in 2019 and percentage change in age standardized DALYs by location and sex

|  | Age-standardized DALYs rate in 2019, (95%UI) | | | Percentage Change in Age-standardized DALYs rate, 1990–2019 | | |
| --- | --- | --- | --- | --- | --- | --- |
|  | Both | Female | Male | Both | Female | Male |
| Sub-Saharan Africa - WB | 715  (634 to 798) | 577  (461 to 697) | 880  (759 to 997) | -19.6 | -17.9 | -20.1 |
| Central Sub-Saharan Africa | 965  (744 to 1321) | 889  (580 to 1466) | 1062  (754 to 1362) | -15.5 | -9.4 | -19.8 |
| Eastern Sub-Saharan Africa | 708  (622 to 802) | 539  (431 to 669) | 907  (763 to 1077) | -25.9 | -23.1 | -26.0 |
| Southern Sub-Saharan Africa | 839  (773 to 908) | 640  (561 to 716) | 1149  (1060 to 1234) | -16.3 | -17.3 | -13.5 |
| Western Sub-Saharan Africa | 619  (543 to 693) | 503  (412 to 583) | 749  (634 to 875) | -16.0 | -17.8 | -15.2 |
| Angola | 729  (597 to 874) | 689  (477 to 896) | 781  (646 to 943) | -33.4 | -30.5 | -35.0 |
| Benin | 693  (581 to 842) | 580  (463 to 725) | 839  (659 to 1051) | -23.8 | -17.7 | -26.1 |
| Botswana | 998  (761 to 1236) | 733  (518 to 935) | 1415  (1089 to 1842) | -23.5 | -15.1 | -26.5 |
| Burkina Faso | 524  (448 to 610) | 447  (358 to 537) | 627  (510 to 767) | -8.2 | -9.1 | -5.0 |
| Burundi | 996  (797 to 1259) | 754  (542 to 1028) | 1239  (915 to 1666) | -29.8 | -25.3 | -35.7 |
| Côte d'Ivoire | 647  (537 to 779) | 490  (389 to 602) | 808  (638 to 988) | -27.5 | -20.5 | -29.7 |
| Cape Verde | 482  (417 to 586) | 399  (328 to 497) | 598  (518 to 759) | -43.3 | -39.8 | -46.4 |
| Cameroon | 714  (564 to 866) | 592  (468 to 727) | 858  (625 to 1073) | -24.0 | -24.7 | -22.8 |
| Central African Republic | 1234  (902 to 1652) | 988  (610 to 1603) | 1550  (1062 to 2038) | -13.1 | -9.7 | -14.5 |
| Chad | 766  (617 to 947) | 625  (473 to 807) | 892  (660 to 1174) | -11.5 | -11.8 | -14.3 |
| Comoros | 631  (521 to 769) | 495  (372 to 665) | 809  (654 to 1001) | -28.4 | -18.3 | -31.8 |
| Congo | 832  (668 to 998) | 803  (559 to 1050) | 869  (538 to 1078) | -33.1 | -21.8 | -43.1 |
| Democratic Republic of the Congo | 1042  (758 to 1493) | 962  (604 to 1682) | 1147  (740 to 1521) | -8.4 | -2.1 | -12.4 |
| Equatorial Guinea | 700  (522 to 994) | 647  (437 to 1201) | 781  (488 to 1020) | -41.4 | -28.3 | -50.6 |
| Eritrea | 841  (652 to 1022) | 656  (411 to 896) | 1134  (827 to 1456) | -20.6 | -10.9 | -29.0 |
| Eswatini | 1093  (882 to 1348) | 689  (496 to 914) | 1788  (1474 to 2174) | -22.2 | -24.8 | -16.6 |
| Ethiopia | 629  (525 to 724) | 489  (380 to 596) | 761  (584 to 939) | -44.8 | -41.4 | -46.9 |
| Gabon | 624  (488 to 758) | 488  (328 to 610) | 794  (551 to 1043) | -31.2 | -30.4 | -32.9 |
| Gambia | 779  (638 to 944) | 649  (501 to 856) | 934  (727 to 1165) | -7.8 | -2.4 | -10.5 |
| Ghana | 670  (473 to 793) | 381  (309 to 448) | 1065  (651 to 1290) | -2.5 | -20.6 | 14.1 |
| Guinea | 810  (660 to 977) | 684  (534 to 880) | 942  (722 to 1177) | -8.0 | -15.0 | -1.7 |
| Guinea-Bissau | 911  (728 to 1115) | 690  (537 to 881) | 1201  (869 to 1527) | -26.7 | -16.3 | -29.5 |
| Kenya | 753  (625 to 933) | 598  (440 to 860) | 954  (726 to 1235) | -3.1 | -11.4 | 7.6 |
| Lesotho | 1677  (1289 to 2156) | 1279  (825 to 1809) | 2330  (1726 to 3037) | 0.0 | 4.0 | -1.8 |
| Liberia | 503  (409 to 635) | 514  (405 to 667) | 496  (365 to 643) | -9.0 | -7.4 | -9.8 |
| Madagascar | 1039  (818 to 1299) | 848  (624 to 1140) | 1262  (949 to 1631) | -7.2 | -1.5 | -7.4 |
| Malawi | 630  (535 to 731) | 442  (342 to 557) | 900  (740 to 1125) | -15.2 | -22.5 | -5.5 |
| Mali | 942  (750 to 1150) | 811  (553 to 1057) | 1066  (834 to 1364) | -8.6 | -8.4 | -10.1 |
| Mauritania | 513  (428 to 608) | 508  (407 to 605) | 517  (417 to 633) | -34.6 | -27.3 | -42.4 |
| Mauritius | 500  (426 to 596) | 304  (252 to 402) | 753  (638 to 881) | -32.3 | -29.1 | -35.3 |
| Mozambique | 659  (534 to 804) | 403  (306 to 524) | 1007  (805 to 1323) | 3.6 | -5.8 | 14.4 |
| Namibia | 1055  (846 to 1284) | 675  (488 to 938) | 1621  (1341 to 1905) | -25.7 | -26.6 | -21.6 |
| Niger | 752  (594 to 991) | 664  (504 to 870) | 846  (605 to 1278) | -17.4 | -11.0 | -22.2 |
| Nigeria | 523  (431 to 623) | 444  (350 to 535) | 605  (455 to 779) | -18.3 | -19.4 | -20.0 |
| Rwanda | 850  (704 to 1038) | 679  (509 to 985) | 1123  (860 to 1420) | -41.5 | -36.7 | -42.4 |
| Sao Tome and Principe | 1409  (1149 to 1657) | 1109  (781 to 1409) | 1750  (1350 to 2227) | 7.3 | 2.4 | 7.5 |
| Senegal | 657  (549 to 779) | 528  (429 to 634) | 803  (647 to 998) | -22.3 | -16.1 | -24.7 |
| Seychelles | 633  (546 to 714) | 401  (324 to 466) | 911  (775 to 1055) | -17.5 | -9.1 | -25.4 |
| Sierra Leone | 716  (578 to 879) | 645  (498 to 830) | 796  (598 to 1039) | -14.9 | 0.2 | -22.9 |
| Somalia | 957  (669 to 1413) | 704  (454 to 1154) | 1350  (887 to 2426) | -21.2 | -12.4 | -20.8 |
| South Africa | 826  (759 to 898) | 633 (560 to 722) | 1128  (1041 to 1232) | -17.3 | -19.4 | -14.4 |
| South Sudan | 644  (493 to 821) | 434  (325 to 593) | 828  (603 to 1092) | -24.9 | -22.1 | -26.3 |
| Sudan | 682  (500 to 898) | 499  (363 to 712) | 836  (540 to 1139) | -19.7 | -20.5 | -20.7 |
| Togo | 713  (584 to 876) | 567  (455 to 728) | 939  (704 to 1194) | -18.1 | -23.1 | -9.1 |
| Uganda | 727  (577 to 899) | 497  (377 to 690) | 1056  (758 to 1337) | -26.1 | -22.9 | -22.5 |
| United Republic of Tanzania | 541  (461 to 625) | 446  (352 to 551) | 656  (534 to 797) | -14.8 | -9.0 | -17.8 |
| Zambia | 720  (600 to 842) | 528  (411 to 671) | 950  (772 to 1138) | -14.7 | -18.0 | -6.8 |
| Zimbabwe | 664  (532 to 787) | 537  (386 to 689) | 854  (662 to 1040) | -3.8 | -1.6 | -1.8 |

**Appendix Table A.4** Percentage of COPD related YLDs attributable to risk factors by sex and location in 2019

|  | **Female** | | | | | **Male** | | | | |
| --- | --- | --- | --- | --- | --- | --- | --- | --- | --- | --- |
|  | **Ambient particulate matter pollution** | **HAP from solid fuels** | **Occupational particulate matter, gases, and fumes** | **Secondhand smoke** | **Smoking** | **Ambient particulate matter pollution** | **HAP from solid fuels** | **Occupational particulate matter, gases, and fumes** | **Secondhand smoke** | **Smoking** |
| **Sub-Saharan Africa - WB** | 12.5 | 37.7 | 12.5 | 5.3 | 9.6 | 14.4 | 31.8 | 16.9 | 4.1 | 35.2 |
| **Central Sub-Saharan Africa** | 9.9 | 44.7 | 12.7 | 3.4 | 5.8 | 11.9 | 36.9 | 15.7 | 2.5 | 33.8 |
| **Eastern Sub-Saharan Africa** | 6.6 | 51.1 | 15.2 | 4.4 | 8.7 | 8.3 | 44.3 | 18.5 | 3.1 | 33.0 |
| **Southern Sub-Saharan Africa** | 15.3 | 8.6 | 7.5 | 8.1 | 22.9 | 16.1 | 6.1 | 13.9 | 5.2 | 51.3 |
| **Western Sub-Saharan Africa** | 16.2 | 39.4 | 13.1 | 5.1 | 5.2 | 18.4 | 33.4 | 17.3 | 4.2 | 27.9 |
| **Angola** | 12.5 | 23.7 | 10.4 | 6.5 | 9.9 | 14.0 | 17.5 | 14.9 | 3.9 | 42.1 |
| **Benin** | 10.2 | 52.4 | 15.4 | 5.1 | 6.6 | 12.9 | 43.1 | 19.0 | 3.9 | 30.6 |
| **Botswana** | 14.0 | 11.5 | 7.5 | 10.0 | 19.3 | 15.0 | 8.2 | 14.4 | 7.4 | 54.8 |
| **Burkina Faso** | 7.3 | 64.1 | 14.6 | 5.8 | 2.6 | 9.8 | 55.9 | 19.6 | 3.8 | 25.6 |
| **Burundi** | 5.1 | 64.8 | 19.1 | 3.2 | 8.3 | 6.9 | 57.6 | 19.5 | 2.5 | 33.2 |
| **Côte d'Ivoire** | 14.5 | 41.9 | 13.3 | 8.4 | 11.3 | 17.5 | 33.3 | 17.2 | 6.3 | 38.7 |
| **Cape Verde** | 24.3 | 9.3 | 10.1 | 5.4 | 7.9 | 25.6 | 6.4 | 14.1 | 4.3 | 27.9 |
| **Cameroon** | 21.0 | 29.7 | 16.6 | 5.0 | 3.9 | 24.0 | 22.2 | 19.0 | 2.8 | 34.2 |
| **Central African Republic** | 7.0 | 64.0 | 11.3 | 4.2 | 5.6 | 9.3 | 55.9 | 16.0 | 3.2 | 32.8 |
| **Chad** | 7.5 | 66.3 | 12.2 | 5.9 | 6.9 | 10.0 | 57.9 | 17.5 | 3.5 | 33.4 |
| **Comoros** | 5.7 | 41.4 | 12.0 | 7.5 | 6.2 | 7.2 | 33.8 | 18.6 | 5.7 | 43.1 |
| **Congo** | 17.9 | 19.5 | 8.3 | 4.8 | 5.0 | 19.3 | 13.8 | 12.0 | 3.5 | 38.3 |
| **Democratic Republic of the Congo** | 8.3 | 53.1 | 14.0 | 2.3 | 4.8 | 10.5 | 44.1 | 16.4 | 2.0 | 31.2 |
| **Equatorial Guinea** | 23.8 | 5.8 | 11.0 | 4.7 | 4.8 | 24.3 | 3.9 | 13.6 | 3.7 | 38.1 |
| **Eritrea** | 12.0 | 42.1 | 14.3 | 4.2 | 1.3 | 14.9 | 34.2 | 18.8 | 4.1 | 32.6 |
| **Eswatini** | 11.7 | 20.4 | 8.8 | 4.4 | 11.1 | 13.2 | 15.1 | 14.7 | 3.0 | 29.9 |
| **Ethiopia** | 6.7 | 56.3 | 11.7 | 2.2 | 1.8 | 8.4 | 50.3 | 17.5 | 2.0 | 18.4 |

| ***Appendix Table A.4 …Continued*** | | | | | | | | | | |
| --- | --- | --- | --- | --- | --- | --- | --- | --- | --- | --- |
|  | **Female** | | | | | **Male** | | | | |
|  | **Ambient particulate matter pollution** | **HAP from solid fuels** | **Occupational particulate matter, gases, and fumes** | **Secondhand smoke** | **Smoking** | **Ambient particulate matter pollution** | **HAP from solid fuels** | **Occupational particulate matter, gases, and fumes** | **Secondhand smoke** | **Smoking** |
| **Gabon** | 21.0 | 2.2 | 6.1 | 5.2 | 5.9 | 21.6 | 1.4 | 10.9 | 3.9 | 34.7 |
| **Gambia** | 13.0 | 49.0 | 9.9 | 9.3 | 3.7 | 16.1 | 39.6 | 15.8 | 6.2 | 42.5 |
| **Ghana** | 20.8 | 22.3 | 17.0 | 3.4 | 5.4 | 23.3 | 16.4 | 19.6 | 2.8 | 23.7 |
| **Guinea** | 8.8 | 60.1 | 12.1 | 6.5 | 6.6 | 11.5 | 51.3 | 17.3 | 4.5 | 45.2 |
| **Guinea-Bissau** | 10.0 | 56.6 | 12.5 | 6.3 | 3.4 | 12.9 | 47.6 | 17.6 | 4.6 | 23.1 |
| **Kenya** | 7.6 | 36.5 | 14.7 | 4.5 | 7.9 | 9.0 | 29.5 | 18.8 | 3.1 | 36.8 |
| **Lesotho** | 11.8 | 28.2 | 9.7 | 11.3 | 11.3 | 13.6 | 21.4 | 17.1 | 6.0 | 58.7 |
| **Liberia** | 10.5 | 52.8 | 12.7 | 4.4 | 6.1 | 13.1 | 43.1 | 16.4 | 3.0 | 28.4 |
| **Madagascar** | 4.6 | 54.0 | 19.5 | 4.8 | 5.0 | 5.9 | 45.5 | 21.7 | 3.4 | 32.2 |
| **Malawi** | 4.8 | 55.8 | 15.4 | 4.6 | 8.2 | 6.4 | 48.8 | 15.6 | 3.2 | 41.5 |
| **Mali** | 8.2 | 66.3 | 13.2 | 5.5 | 5.8 | 10.8 | 57.6 | 18.0 | 3.9 | 35.2 |
| **Mauritania** | 23.4 | 23.6 | 9.0 | 5.8 | 10.2 | 25.8 | 17.0 | 13.0 | 4.0 | 39.6 |
| **Mauritius** | 9.1 | 0.3 | 7.5 | 12.3 | 9.6 | 9.4 | 0.2 | 16.1 | 6.8 | 54.9 |
| **Mozambique** | 3.7 | 61.6 | 17.2 | 4.4 | 9.2 | 5.0 | 55.2 | 17.4 | 3.1 | 36.3 |
| **Namibia** | 12.8 | 15.6 | 7.1 | 5.8 | 25.8 | 14.2 | 11.4 | 13.2 | 4.5 | 42.5 |
| **Niger** | 6.9 | 72.9 | 12.3 | 5.4 | 2.8 | 9.4 | 65.6 | 19.5 | 3.9 | 22.9 |
| **Nigeria** | 20.3 | 27.5 | 12.0 | 4.1 | 4.0 | 22.1 | 23.5 | 15.5 | 4.3 | 19.1 |
| **Rwanda** | 9.3 | 47.8 | 18.7 | 5.0 | 24.6 | 11.8 | 39.6 | 20.1 | 3.7 | 47.8 |
| **Sao Tome and Principe** | 13.2 | 27.8 | 5.6 | 2.6 | 7.3 | 15.1 | 20.9 | 14.2 | 2.1 | 29.9 |
| **Senegal** | 14.0 | 45.7 | 8.8 | 9.6 | 3.8 | 17.3 | 37.0 | 17.9 | 7.2 | 35.9 |
| **Seychelles** | 9.6 | 0.1 | 9.9 | 11.0 | 14.2 | 9.9 | 0.1 | 14.9 | 6.6 | 61.2 |
| **Sierra Leone** | 9.4 | 57.5 | 13.1 | 7.5 | 10.9 | 12.0 | 48.1 | 15.8 | 4.7 | 41.0 |
| **Somalia** | 1.9 | 80.4 | 13.4 | 5.0 | 6.3 | 2.7 | 77.1 | 19.9 | 3.7 | 36.4 |
| **South Africa** | 16.7 | 3.3 | 6.2 | 7.9 | 24.9 | 17.2 | 2.4 | 13.1 | 5.4 | 51.2 |
| **South Sudan** | 8.0 | 53.4 | 15.1 | 5.2 | 6.2 | 10.4 | 45.3 | 17.1 | 3.8 | 35.8 |
| **Sudan** | 22.1 | 18.7 | 5.3 | 10.7 | 8.8 | 24.5 | 13.6 | 16.3 | 9.0 | 54.4 |
| **Togo** | 11.9 | 45.4 | 16.1 | 6.2 | 11.0 | 14.8 | 36.9 | 18.4 | 4.7 | 41.0 |
| **Uganda** | 8.4 | 50.3 | 15.9 | 3.9 | 9.4 | 10.8 | 42.4 | 18.6 | 3.1 | 30.7 |
| **United Republic of Tanzania** | 6.4 | 49.0 | 16.0 | 6.8 | 16.5 | 8.3 | 41.0 | 19.3 | 4.1 | 42.4 |
| **Zambia** | 9.6 | 35.0 | 12.8 | 5.7 | 13.1 | 11.5 | 27.6 | 16.9 | 3.5 | 37.7 |
| **Zimbabwe** | 7.8 | 36.2 | 15.2 | 8.7 | 14.7 | 9.4 | 28.6 | 19.4 | 3.9 | 55.1 |

*Notes: YLDs - years lived with disabilities; COPD - chronic obstructive pulmonary disease; HAP – household air pollution*

**Appendix Table A.5** Percentage of COPD related Deaths attributable to risk factors by sex and location in 2019

|  | **Female** | | | | | | | **Male** | | | | | | |
| --- | --- | --- | --- | --- | --- | --- | --- | --- | --- | --- | --- | --- | --- | --- |
|  | **Ambient ozone pollution** | **Ambient particulate matter pollution** | **HAP from solid fuels** | **Low temperature** | **Occupational particulate matter, gases, and fumes** | **Secondhand smoke** | **Smoking** | **Ambient ozone pollution** | **Ambient particulate matter pollution** | **HAP from solid fuels** | **Low temperature** | **Occupational particulate matter, gases, and fumes** | **Secondhand smoke** | **Smoking** |
| **Sub-Saharan Africa** | 7.5 | 12.6 | 42.2 | 4.9 | 14.3 | 4.3 | 12.1 | 7.6 | 15.0 | 35.9 | 4.6 | 19.3 | 3.9 | 39.1 |
| **Central Sub-Saharan Africa** | 8.6 | 10.0 | 50.0 | 3.1 | 14.6 | 2.5 | 6.0 | 8.6 | 12.4 | 40.5 | 3.1 | 18.1 | 2.0 | 37.7 |
| **Eastern Sub-Saharan Africa** | 5.8 | 7.1 | 56.4 | 5.6 | 17.4 | 3.3 | 12.8 | 5.9 | 8.8 | 48.7 | 5.5 | 20.8 | 2.7 | 39.5 |
| **Southern Sub-Saharan Africa** | 5.3 | 16.4 | 8.7 | 12.4 | 8.1 | 7.3 | 28.2 | 5.2 | 16.9 | 6.0 | 12.3 | 15.0 | 5.5 | 55.2 |
| **Western Sub-Saharan Africa** | 9.8 | 16.8 | 43.7 | 1.4 | 15.2 | 4.4 | 6.5 | 9.9 | 19.7 | 36.7 | 1.3 | 20.1 | 4.2 | 30.1 |
| **Angola** | 6.8 | 13.3 | 25.3 | 3.9 | 12.4 | 5.0 | 10.7 | 6.8 | 14.9 | 18.6 | 3.9 | 17.4 | 3.2 | 48.8 |
| **Benin** | 11.5 | 10.8 | 55.3 | 0.7 | 17.5 | 4.0 | 7.0 | 11.6 | 13.7 | 45.8 | 0.7 | 21.6 | 3.3 | 37.8 |
| **Botswana** | 5.9 | 14.8 | 12.2 | 4.6 | 8.9 | 9.8 | 26.9 | 5.9 | 15.6 | 8.5 | 4.6 | 15.7 | 7.9 | 61.2 |
| **Burkina Faso** | 8.5 | 7.8 | 68.4 | 1.6 | 16.2 | 5.1 | 3.1 | 8.6 | 10.6 | 60.4 | 1.6 | 22.1 | 3.9 | 26.0 |
| **Burundi** | 8.0 | 5.4 | 69.5 | 7.3 | 21.7 | 2.2 | 8.8 | 8.0 | 7.3 | 61.0 | 7.3 | 21.9 | 2.0 | 40.1 |
| **Côte d'Ivoire** | 7.1 | 15.5 | 44.8 | 0.5 | 15.2 | 7.6 | 16.7 | 7.1 | 18.7 | 35.6 | 0.5 | 19.5 | 6.5 | 38.0 |
| **Cape Verde** | 4.6 | 26.2 | 10.0 | 3.9 | 11.6 | 4.4 | 9.3 | 4.6 | 27.5 | 6.9 | 3.9 | 16.2 | 4.1 | 32.0 |
| **Cameroon** | 10.2 | 22.1 | 31.2 | 2.0 | 18.5 | 3.9 | 5.8 | 10.2 | 25.3 | 23.4 | 2.0 | 21.1 | 2.5 | 34.3 |
| **Central African Republic** | 12.1 | 7.4 | 68.1 | 0.9 | 13.2 | 3.4 | 6.8 | 12.2 | 9.8 | 59.2 | 0.9 | 18.2 | 2.9 | 38.3 |
| **Chad** | 8.7 | 8.0 | 70.6 | 3.8 | 13.9 | 4.9 | 9.9 | 8.8 | 10.7 | 62.1 | 3.8 | 19.6 | 3.4 | 40.6 |
| **Comoros** | 1.8 | 6.2 | 44.9 | 0.8 | 13.7 | 6.3 | 9.6 | 1.8 | 7.6 | 35.9 | 0.8 | 20.9 | 5.4 | 56.6 |
| **Congo** | 7.7 | 18.8 | 20.5 | 1.1 | 9.5 | 3.9 | 5.0 | 7.7 | 20.5 | 14.7 | 1.1 | 13.9 | 3.1 | 46.7 |
| **Democratic Republic of the Congo** | 9.0 | 8.8 | 56.2 | 3.3 | 15.5 | 1.8 | 5.2 | 9.0 | 11.1 | 46.6 | 3.3 | 18.7 | 1.6 | 34.9 |
| **Equatorial Guinea** | 6.4 | 25.2 | 6.1 | 1.6 | 12.8 | 4.0 | 5.4 | 6.4 | 25.8 | 4.1 | 1.6 | 16.0 | 3.2 | 42.5 |
| **Eritrea** | 7.0 | 12.9 | 45.3 | 2.5 | 16.2 | 3.3 | 1.8 | 7.0 | 15.7 | 36.1 | 2.5 | 21.0 | 3.9 | 32.5 |
| **Eswatini** | 5.0 | 12.4 | 21.7 | 9.6 | 10.2 | 3.6 | 23.1 | 5.0 | 13.6 | 15.6 | 9.6 | 16.2 | 3.0 | 34.2 |
| **Ethiopia** | 7.7 | 7.0 | 63.2 | 7.4 | 14.2 | 1.5 | 2.1 | 7.6 | 8.8 | 55.3 | 7.1 | 20.2 | 1.5 | 23.9 |

| ***Appendix Table A.5 … Continued*** | | | | | | | | | | | | | | | |
| --- | --- | --- | --- | --- | --- | --- | --- | --- | --- | --- | --- | --- | --- | --- | --- |
|  | **Female** | | | | | | | | **Male** | | | | | | |
|  | **Ambient ozone pollution** | **Ambient particulate matter pollution** | **HAP from solid fuels** | **Low temperature** | **Occupational particulate matter, gases, and fumes** | **Secondhand smoke** | **Smoking** | **Ambient ozone pollution** | | **Ambient particulate matter pollution** | **HAP from solid fuels** | **Low temperature** | **Occupational particulate matter, gases, and fumes** | **Secondhand smoke** | **Smoking** |
| **Gabon** | 3.5 | 22.6 | 2.3 | 1.0 | 7.1 | 4.0 | 6.5 | 3.5 | | 22.8 | 1.5 | 1.0 | 12.9 | 3.5 | 38.8 |
| **Gambia** | 6.9 | 13.8 | 51.8 | 1.0 | 11.4 | 9.0 | 5.2 | 6.9 | | 17.1 | 42.1 | 1.0 | 17.7 | 6.5 | 46.5 |
| **Ghana** | 10.0 | 22.3 | 23.9 | 0.4 | 21.0 | 2.5 | 7.6 | 10.0 | | 24.8 | 17.4 | 0.4 | 23.1 | 2.1 | 30.7 |
| **Guinea** | 7.9 | 9.3 | 63.5 | 0.9 | 13.5 | 5.6 | 9.0 | 7.9 | | 12.1 | 54.3 | 0.9 | 18.9 | 4.3 | 49.2 |
| **Guinea-Bissau** | 7.0 | 10.6 | 59.9 | 0.8 | 14.1 | 5.4 | 4.0 | 7.1 | | 13.6 | 50.3 | 0.8 | 19.4 | 4.4 | 24.3 |
| **Kenya** | 5.1 | 8.4 | 39.4 | 7.3 | 17.0 | 3.6 | 12.9 | 4.8 | | 9.5 | 31.9 | 7.2 | 21.1 | 3.1 | 44.8 |
| **Lesotho** | 6.4 | 12.4 | 29.6 | 20.9 | 11.0 | 9.4 | 21.3 | 6.4 | | 14.1 | 22.2 | 20.9 | 18.7 | 6.3 | 63.7 |
| **Liberia** | 5.7 | 11.1 | 56.2 | 0.6 | 14.8 | 3.8 | 7.3 | 5.7 | | 14.2 | 46.7 | 0.6 | 19.1 | 2.8 | 32.7 |
| **Madagascar** | 3.0 | 4.8 | 56.9 | 5.0 | 20.9 | 3.9 | 6.4 | 3.1 | | 6.2 | 47.8 | 5.0 | 23.3 | 3.1 | 36.3 |
| **Malawi** | 4.7 | 5.2 | 60.9 | 5.3 | 18.5 | 3.1 | 12.1 | 4.7 | | 6.8 | 51.7 | 5.3 | 17.0 | 2.7 | 56.4 |
| **Mali** | 7.2 | 8.5 | 69.5 | 4.2 | 14.4 | 4.8 | 6.9 | 7.2 | | 11.4 | 60.6 | 4.2 | 19.5 | 3.8 | 42.3 |
| **Mauritania** | 5.3 | 24.8 | 25.0 | 4.8 | 11.4 | 5.0 | 7.9 | 5.3 | | 27.7 | 18.3 | 4.8 | 13.9 | 3.8 | 40.7 |
| **Mauritius** | 0.0 | 9.8 | 0.3 | 2.3 | 7.4 | 11.8 | 12.1 | 0.0 | | 9.8 | 0.2 | 2.3 | 16.4 | 7.6 | 62.6 |
| **Mozambique** | 3.6 | 4.0 | 67.1 | 2.9 | 19.9 | 3.5 | 12.7 | 3.6 | | 5.3 | 58.5 | 2.9 | 19.9 | 2.7 | 46.5 |
| **Namibia** | 7.0 | 13.7 | 16.7 | 5.9 | 8.4 | 5.1 | 43.6 | 7.0 | | 14.7 | 11.8 | 5.9 | 14.6 | 4.6 | 52.0 |
| **Niger** | 9.0 | 7.3 | 77.4 | 4.1 | 12.7 | 4.5 | 3.0 | 9.1 | | 10.1 | 70.2 | 4.1 | 21.1 | 3.4 | 27.7 |
| **Nigeria** | 11.9 | 21.7 | 30.8 | 0.6 | 15.0 | 3.3 | 5.1 | 11.8 | | 23.4 | 29.1 | 0.7 | 19.4 | 4.5 | 21.3 |
| **Rwanda** | 8.9 | 10.0 | 51.1 | 9.7 | 20.9 | 3.3 | 42.5 | 8.9 | | 12.4 | 41.7 | 9.7 | 21.9 | 3.4 | 61.5 |
| **Sao Tome and Principe** | 5.6 | 13.7 | 28.9 | 0.5 | 6.6 | 1.9 | 8.3 | 5.6 | | 15.6 | 21.6 | 0.5 | 15.7 | 1.6 | 30.5 |
| **Senegal** | 6.3 | 15.0 | 48.9 | 1.3 | 10.1 | 9.3 | 3.9 | 6.3 | | 18.4 | 39.3 | 1.3 | 19.9 | 7.6 | 35.1 |
| **Seychelles** | 1.7 | 10.3 | 0.1 | 0.3 | 10.7 | 9.7 | 18.3 | 1.7 | | 10.3 | 0.1 | 0.3 | 15.6 | 7.5 | 70.3 |
| **Sierra Leone** | 6.2 | 9.9 | 60.7 | 0.6 | 14.9 | 7.5 | 9.9 | 6.2 | | 12.8 | 51.4 | 0.6 | 18.6 | 5.3 | 46.7 |
| **Somalia** | 4.2 | 2.0 | 86.8 | 1.4 | 15.1 | 4.2 | 10.3 | 4.2 | | 2.9 | 82.1 | 1.4 | 22.2 | 3.8 | 45.7 |
| **South Africa** | 5.0 | 17.7 | 3.8 | 13.1 | 6.9 | 7.2 | 28.6 | 5.0 | | 17.9 | 2.5 | 13.0 | 14.2 | 5.6 | 54.3 |
| **South Sudan** | 6.8 | 8.7 | 57.6 | 0.7 | 17.3 | 4.8 | 10.3 | 6.8 | | 11.1 | 48.3 | 0.7 | 19.4 | 4.0 | 45.3 |
| **Sudan** | 8.6 | 23.4 | 19.9 | 3.7 | 5.7 | 9.7 | 9.7 | 8.7 | | 25.7 | 14.3 | 3.7 | 17.9 | 9.5 | 58.9 |
| **Togo** | 11.3 | 12.7 | 48.3 | 0.5 | 18.1 | 4.5 | 14.7 | 11.3 | | 15.6 | 38.9 | 0.5 | 20.3 | 4.0 | 47.6 |
| **Uganda** | 6.8 | 9.0 | 54.4 | 3.1 | 18.1 | 2.8 | 16.6 | 6.8 | | 11.4 | 44.9 | 3.1 | 20.9 | 2.8 | 37.3 |
| **United Republic of Tanzania** | 4.3 | 7.0 | 53.2 | 4.6 | 18.8 | 5.4 | 23.1 | 4.3 | | 8.9 | 44.0 | 4.6 | 22.0 | 4.0 | 53.0 |
| **Zambia** | 6.9 | 10.3 | 37.7 | 4.6 | 14.6 | 4.7 | 26.2 | 6.9 | | 12.2 | 29.2 | 4.6 | 18.9 | 3.4 | 47.4 |
| **Zimbabwe** | 6.0 | 8.4 | 38.8 | 5.7 | 17.6 | 6.7 | 26.8 | 6.0 | | 9.9 | 30.0 | 5.7 | 21.2 | 3.9 | 64.5 |

*Notes: COPD - chronic obstructive pulmonary disease; HAP – household air pollution*

**Appendix Table A.6** Percentage of COPD related YLLs attributable to risk factors by sex and location in 2019

|  | **Female** | | | | | | | **Male** | | | | | | |
| --- | --- | --- | --- | --- | --- | --- | --- | --- | --- | --- | --- | --- | --- | --- |
|  | **Ambient ozone pollution** | **Ambient particulate matter pollution** | **HAP from solid fuels** | **Low temperature** | **Occupational particulate matter, gases, and fumes** | **Secondhand smoke** | **Smoking** | **Ambient ozone pollution** | **Ambient particulate matter pollution** | **HAP from solid fuels** | **Low temperature** | **Occupational particulate matter, gases, and fumes** | **Secondhand smoke** | **Smoking** |
| **Sub-Saharan Africa** | 7.5 | 12.2 | 42.6 | 4.7 | 14.3 | 4.6 | 11.4 | 7.5 | 14.5 | 36.4 | 4.6 | 19.4 | 4.0 | 39.9 |
| **Central Sub-Saharan Africa** | 8.6 | 9.9 | 49.5 | 3.1 | 14.6 | 2.7 | 6.6 | 8.6 | 12.2 | 40.5 | 3.1 | 18.0 | 2.2 | 39.0 |
| **Eastern Sub-Saharan Africa** | 5.7 | 6.9 | 55.3 | 5.6 | 17.2 | 3.7 | 11.6 | 5.8 | 8.7 | 48.3 | 5.4 | 20.9 | 2.9 | 39.1 |
| **Southern Sub-Saharan Africa** | 5.3 | 16.5 | 8.7 | 12.4 | 8.3 | 7.7 | 28.2 | 5.3 | 16.9 | 6.3 | 12.2 | 15.2 | 5.5 | 56.2 |
| **Western Sub-Saharan Africa** | 9.6 | 16.3 | 43.6 | 1.4 | 14.8 | 4.6 | 6.5 | 9.7 | 19.2 | 37.5 | 1.4 | 20.1 | 4.3 | 32.1 |
| **Angola** | 6.7 | 13.1 | 25.0 | 3.9 | 12.1 | 5.5 | 11.6 | 6.7 | 14.8 | 18.5 | 3.9 | 17.3 | 3.5 | 50.0 |
| **Benin** | 11.2 | 10.5 | 53.8 | 0.7 | 17.0 | 4.4 | 7.8 | 11.4 | 13.5 | 45.3 | 0.7 | 21.5 | 3.6 | 37.5 |
| **Botswana** | 5.8 | 14.7 | 12.1 | 4.6 | 8.7 | 10.0 | 25.2 | 5.8 | 15.5 | 8.4 | 4.6 | 15.6 | 7.7 | 59.8 |
| **Burkina Faso** | 8.0 | 7.4 | 64.7 | 1.6 | 15.6 | 5.1 | 3.0 | 8.5 | 10.5 | 59.8 | 1.6 | 22.2 | 4.0 | 29.0 |
| **Burundi** | 7.8 | 5.3 | 67.8 | 7.3 | 21.3 | 2.5 | 9.2 | 8.0 | 7.2 | 60.4 | 7.3 | 21.9 | 2.2 | 40.0 |
| **Côte d'Ivoire** | 6.9 | 15.1 | 43.8 | 0.5 | 15.0 | 7.9 | 15.9 | 7.0 | 18.5 | 35.2 | 0.5 | 19.5 | 6.5 | 41.8 |
| **Cape Verde** | 4.6 | 25.8 | 9.9 | 3.9 | 11.8 | 4.7 | 10.5 | 4.6 | 27.3 | 6.9 | 3.9 | 16.4 | 4.3 | 34.5 |
| **Cameroon** | 10.0 | 21.6 | 30.6 | 2.0 | 18.1 | 4.3 | 4.6 | 10.1 | 25.1 | 23.2 | 2.0 | 21.0 | 2.7 | 37.6 |
| **Central African Republic** | 12.0 | 7.3 | 67.1 | 0.9 | 12.9 | 3.7 | 6.9 | 12.1 | 9.8 | 58.7 | 0.9 | 18.1 | 3.1 | 38.6 |
| **Chad** | 8.5 | 7.8 | 68.9 | 3.8 | 13.7 | 5.2 | 8.9 | 8.6 | 10.6 | 61.3 | 3.8 | 19.7 | 3.5 | 39.7 |
| **Comoros** | 1.8 | 6.1 | 44.1 | 0.8 | 13.6 | 6.8 | 8.8 | 1.8 | 7.6 | 35.7 | 0.8 | 21.0 | 5.6 | 53.5 |
| **Congo** | 7.6 | 18.7 | 20.4 | 1.1 | 9.4 | 4.2 | 5.7 | 7.6 | 20.4 | 14.6 | 1.1 | 13.9 | 3.3 | 45.6 |
| **Democratic Republic of the Congo** | 8.9 | 8.7 | 55.7 | 3.3 | 15.5 | 2.0 | 5.6 | 8.9 | 11.0 | 46.3 | 3.3 | 18.6 | 1.8 | 36.4 |
| **Equatorial Guinea** | 6.3 | 25.0 | 6.0 | 1.6 | 12.8 | 4.2 | 5.6 | 6.3 | 25.7 | 4.1 | 1.6 | 16.0 | 3.5 | 44.4 |
| **Eritrea** | 6.9 | 12.7 | 44.4 | 2.5 | 16.0 | 3.6 | 1.7 | 6.9 | 15.6 | 35.7 | 2.5 | 20.9 | 4.1 | 34.8 |
| **Eswatini** | 4.9 | 12.3 | 21.6 | 9.6 | 10.2 | 3.9 | 18.6 | 4.9 | 13.6 | 15.6 | 9.6 | 16.1 | 3.0 | 33.5 |
| **Ethiopia** | 7.5 | 6.9 | 61.9 | 7.3 | 14.1 | 1.7 | 2.2 | 7.5 | 8.8 | 54.7 | 7.1 | 20.3 | 1.7 | 23.6 |

| ***Appendix Table A.6 … Continued*** | | | | | | | | | | | | | | |  |
| --- | --- | --- | --- | --- | --- | --- | --- | --- | --- | --- | --- | --- | --- | --- | --- |
|  | **Female** | | | | | | | **Male** | | | | | | | |
|  | **Ambient ozone pollution** | **Ambient particulate matter pollution** | **HAP from solid fuels** | **Low temperature** | **Occupational particulate matter, gases, and fumes** | **Secondhand smoke** | **Smoking** | **Ambient ozone pollution** | **Ambient particulate matter pollution** | **HAP from solid fuels** | **Low temperature** | **Occupational particulate matter, gases, and fumes** | **Secondhand smoke** | **Smoking** | |
| **Gabon** | 3.4 | 22.5 | 2.3 | 1.0 | 7.2 | 4.4 | 7.0 | 3.4 | 22.7 | 1.5 | 1.0 | 12.8 | 3.7 | 40.2 | |
| **Gambia** | 6.8 | 13.6 | 51.1 | 1.0 | 11.2 | 9.1 | 4.9 | 6.8 | 16.9 | 41.6 | 1.0 | 17.7 | 6.5 | 48.1 | |
| **Ghana** | 9.8 | 21.9 | 23.4 | 0.4 | 20.2 | 2.8 | 6.8 | 9.9 | 24.5 | 17.2 | 0.4 | 22.6 | 2.5 | 29.2 | |
| **Guinea** | 7.7 | 9.0 | 61.7 | 0.9 | 13.2 | 5.9 | 8.5 | 7.8 | 12.0 | 53.6 | 0.9 | 18.9 | 4.5 | 50.5 | |
| **Guinea-Bissau** | 6.9 | 10.4 | 58.9 | 0.8 | 13.8 | 5.8 | 4.1 | 7.0 | 13.4 | 49.7 | 0.8 | 19.4 | 4.5 | 25.8 | |
| **Kenya** | 5.0 | 8.3 | 38.6 | 7.3 | 16.8 | 4.0 | 11.4 | 4.8 | 9.4 | 31.7 | 7.2 | 21.1 | 3.1 | 44.2 | |
| **Lesotho** | 6.4 | 12.3 | 29.4 | 20.9 | 10.9 | 10.3 | 16.6 | 6.4 | 14.0 | 22.0 | 20.9 | 18.5 | 6.2 | 63.4 | |
| **Liberia** | 5.6 | 10.9 | 54.8 | 0.6 | 14.3 | 4.0 | 7.7 | 5.7 | 14.0 | 46.3 | 0.6 | 19.0 | 2.9 | 35.2 | |
| **Madagascar** | 3.0 | 4.7 | 55.4 | 5.0 | 20.7 | 4.2 | 5.8 | 3.0 | 6.1 | 47.1 | 5.0 | 23.3 | 3.3 | 35.6 | |
| **Malawi** | 4.6 | 5.1 | 59.5 | 5.3 | 18.0 | 3.6 | 12.7 | 4.6 | 6.7 | 51.3 | 5.3 | 17.2 | 3.0 | 52.3 | |
| **Mali** | 7.1 | 8.4 | 68.5 | 4.2 | 14.3 | 5.1 | 7.2 | 7.2 | 11.3 | 60.1 | 4.2 | 19.5 | 3.9 | 40.3 | |
| **Mauritania** | 5.3 | 24.6 | 24.7 | 4.8 | 10.9 | 5.3 | 9.4 | 5.3 | 27.4 | 18.1 | 4.8 | 14.4 | 4.0 | 43.7 | |
| **Mauritius** | 0.0 | 9.5 | 0.3 | 2.3 | 7.8 | 12.0 | 11.5 | 0.0 | 9.7 | 0.2 | 2.3 | 17.0 | 7.3 | 60.7 | |
| **Mozambique** | 3.5 | 3.9 | 65.4 | 2.9 | 19.6 | 3.7 | 12.6 | 3.6 | 5.3 | 58.1 | 2.9 | 19.8 | 2.9 | 44.7 | |
| **Namibia** | 7.0 | 13.7 | 16.6 | 5.9 | 8.4 | 5.4 | 38.7 | 7.0 | 14.7 | 11.7 | 5.9 | 14.5 | 4.6 | 48.1 | |
| **Niger** | 8.7 | 7.1 | 75.1 | 4.1 | 12.8 | 4.7 | 3.2 | 9.0 | 10.0 | 69.4 | 4.1 | 21.4 | 3.7 | 25.8 | |
| **Nigeria** | 11.6 | 21.2 | 31.2 | 0.6 | 14.5 | 3.5 | 5.3 | 11.6 | 23.0 | 30.6 | 0.7 | 19.5 | 4.7 | 23.5 | |
| **Rwanda** | 8.6 | 9.7 | 49.7 | 9.7 | 20.6 | 3.9 | 36.7 | 8.8 | 12.3 | 41.3 | 9.7 | 22.1 | 3.5 | 58.2 | |
| **Sao Tome and Principe** | 5.6 | 13.5 | 28.6 | 0.5 | 6.3 | 2.2 | 9.0 | 5.6 | 15.4 | 21.4 | 0.5 | 15.5 | 1.8 | 32.8 | |
| **Senegal** | 6.2 | 14.7 | 48.1 | 1.3 | 10.0 | 9.4 | 4.6 | 6.2 | 18.2 | 38.9 | 1.3 | 19.9 | 7.5 | 39.5 | |
| **Seychelles** | 1.7 | 10.2 | 0.1 | 0.3 | 11.0 | 10.3 | 17.7 | 1.7 | 10.3 | 0.1 | 0.3 | 16.0 | 7.2 | 67.8 | |
| **Sierra Leone** | 6.0 | 9.6 | 59.0 | 0.6 | 14.5 | 7.4 | 11.6 | 6.1 | 12.6 | 50.7 | 0.6 | 18.3 | 5.1 | 47.9 | |
| **Somalia** | 4.1 | 2.0 | 84.8 | 1.4 | 15.0 | 4.5 | 9.2 | 4.2 | 2.9 | 81.3 | 1.4 | 22.2 | 3.7 | 44.6 | |
| **South Africa** | 5.1 | 17.9 | 3.3 | 13.1 | 7.1 | 7.6 | 29.6 | 5.1 | 18.1 | 2.4 | 13.0 | 14.4 | 5.6 | 55.7 | |
| **South Sudan** | 6.6 | 8.4 | 55.7 | 0.7 | 16.9 | 4.8 | 9.3 | 6.8 | 11.0 | 47.9 | 0.7 | 19.5 | 3.9 | 44.6 | |
| **Sudan** | 8.4 | 22.6 | 19.2 | 3.7 | 5.7 | 10.1 | 9.9 | 8.4 | 25.0 | 13.9 | 3.7 | 17.6 | 9.3 | 57.7 | |
| **Togo** | 11.1 | 12.5 | 47.4 | 0.5 | 17.8 | 5.1 | 14.8 | 11.2 | 15.5 | 38.5 | 0.5 | 20.3 | 4.4 | 47.5 | |
| **Uganda** | 6.6 | 8.8 | 53.2 | 3.1 | 17.9 | 3.1 | 14.5 | 6.7 | 11.3 | 44.5 | 3.1 | 20.8 | 3.0 | 36.8 | |
| **United Republic of Tanzania** | 4.2 | 6.8 | 51.4 | 4.6 | 18.2 | 5.9 | 21.6 | 4.3 | 8.8 | 43.6 | 4.6 | 21.9 | 4.0 | 52.3 | |
| **Zambia** | 6.8 | 10.1 | 37.1 | 4.6 | 14.4 | 5.1 | 21.0 | 6.8 | 12.1 | 29.0 | 4.6 | 19.0 | 3.4 | 46.4 | |
| **Zimbabwe** | 6.0 | 8.3 | 38.6 | 5.7 | 17.6 | 7.2 | 24.1 | 6.0 | 9.9 | 29.8 | 5.7 | 21.2 | 3.9 | 63.8 | |

*Notes: YLLs - years life lost; COPD - chronic obstructive pulmonary disease; HAP – household air pollution*

**Appendix Table A.7** Percentage of COPD related DALYs attributable to risk factors by sex and location in 2019

|  | **Female** | | | | | | | **Male** | | | | | | |
| --- | --- | --- | --- | --- | --- | --- | --- | --- | --- | --- | --- | --- | --- | --- |
|  | **Ambient ozone pollution** | **Ambient particulate matter pollution** | **HAP from solid fuels** | **Low temperature** | **Occupational particulate matter, gases, and fumes** | **Secondhand smoke** | **Smoking** | **Ambient ozone pollution** | **Ambient particulate matter pollution** | **HAP from solid fuels** | **Low temperature** | **Occupational particulate matter, gases, and fumes** | **Secondhand smoke** | **Smoking** |
| **Sub-Saharan Africa** | 4.7 | 12.3 | 40.9 | 3.0 | 13.6 | 4.8 | 10.7 | 5.8 | 14.5 | 35.4 | 3.5 | 18.8 | 4.0 | 38.8 |
| **Central Sub-Saharan Africa** | 5.9 | 9.9 | 48.1 | 2.2 | 14.0 | 2.9 | 6.3 | 6.9 | 12.2 | 39.9 | 2.5 | 17.6 | 2.3 | 38.0 |
| **Eastern Sub-Saharan Africa** | 3.7 | 6.8 | 53.9 | 3.6 | 16.5 | 3.9 | 10.6 | 4.6 | 8.6 | 47.5 | 4.3 | 20.4 | 2.9 | 37.9 |
| **Southern Sub-Saharan Africa** | 3.0 | 16.0 | 8.7 | 7.0 | 8.0 | 7.9 | 25.9 | 3.9 | 16.7 | 6.2 | 8.9 | 14.8 | 5.4 | 54.9 |
| **Western Sub-Saharan Africa** | 5.7 | 16.2 | 41.9 | 0.8 | 14.1 | 4.8 | 6.0 | 7.4 | 19.0 | 36.5 | 1.1 | 19.4 | 4.3 | 31.0 |
| **Angola** | 4.3 | 12.9 | 24.5 | 2.5 | 11.5 | 5.9 | 11.0 | 5.1 | 14.6 | 18.3 | 3.0 | 16.7 | 3.6 | 48.1 |
| **Benin** | 6.4 | 10.4 | 53.2 | 0.4 | 16.3 | 4.7 | 7.3 | 8.5 | 13.4 | 44.7 | 0.5 | 20.8 | 3.7 | 35.8 |
| **Botswana** | 3.3 | 14.4 | 11.9 | 2.7 | 8.2 | 10.0 | 22.7 | 4.6 | 15.4 | 8.3 | 3.7 | 15.3 | 7.6 | 58.8 |
| **Burkina Faso** | 4.7 | 7.4 | 64.4 | 0.9 | 15.2 | 5.4 | 2.8 | 6.3 | 10.3 | 58.8 | 1.1 | 21.5 | 3.9 | 28.1 |
| **Burundi** | 5.6 | 5.2 | 66.9 | 5.2 | 20.6 | 2.7 | 9.0 | 6.6 | 7.1 | 59.9 | 6.0 | 21.4 | 2.3 | 38.8 |
| **Côte d'Ivoire** | 4.0 | 14.8 | 43.0 | 0.3 | 14.3 | 8.1 | 13.9 | 5.3 | 18.3 | 34.7 | 0.4 | 18.9 | 6.5 | 41.0 |
| **Cape Verde** | 2.3 | 25.1 | 9.6 | 2.0 | 11.0 | 5.0 | 9.2 | 3.2 | 26.8 | 6.7 | 2.8 | 15.7 | 4.3 | 32.5 |
| **Cameroon** | 5.2 | 21.4 | 30.2 | 1.0 | 17.4 | 4.6 | 4.3 | 7.2 | 24.7 | 22.9 | 1.4 | 20.4 | 2.7 | 36.7 |
| **Central African Republic** | 8.9 | 7.2 | 66.3 | 0.7 | 12.5 | 3.8 | 6.5 | 10.3 | 9.7 | 58.3 | 0.8 | 17.8 | 3.1 | 37.8 |
| **Chad** | 5.6 | 7.7 | 68.0 | 2.5 | 13.2 | 5.4 | 8.2 | 6.8 | 10.4 | 60.6 | 2.9 | 19.2 | 3.5 | 38.4 |
| **Comoros** | 1.1 | 6.0 | 43.1 | 0.5 | 13.0 | 7.1 | 7.9 | 1.4 | 7.5 | 35.2 | 0.6 | 20.5 | 5.6 | 51.1 |
| **Congo** | 4.6 | 18.4 | 20.0 | 0.6 | 9.0 | 4.5 | 5.4 | 6.0 | 20.2 | 14.4 | 0.8 | 13.5 | 3.4 | 44.0 |
| **Democratic Republic of the Congo** | 6.3 | 8.6 | 54.9 | 2.3 | 15.1 | 2.1 | 5.3 | 7.2 | 10.9 | 45.9 | 2.6 | 18.2 | 1.8 | 35.4 |
| **Equatorial Guinea** | 3.6 | 24.5 | 5.9 | 0.9 | 12.0 | 4.4 | 5.3 | 4.9 | 25.4 | 4.0 | 1.3 | 15.4 | 3.5 | 43.0 |
| **Eritrea** | 4.6 | 12.5 | 43.7 | 1.7 | 15.5 | 3.8 | 1.6 | 5.4 | 15.4 | 35.4 | 2.0 | 20.4 | 4.1 | 34.3 |
| **Eswatini** | 2.8 | 12.1 | 21.1 | 5.5 | 9.6 | 4.1 | 15.4 | 3.9 | 13.5 | 15.5 | 7.6 | 15.8 | 3.0 | 32.8 |
| **Ethiopia** | 5.2 | 6.8 | 60.2 | 5.0 | 13.3 | 1.8 | 2.1 | 6.1 | 8.7 | 53.9 | 5.8 | 19.8 | 1.8 | 22.7 |

| ***Appendix Table A.7 … Continued*** | | | | | | | | | | | | | | |
| --- | --- | --- | --- | --- | --- | --- | --- | --- | --- | --- | --- | --- | --- | --- |
|  | **Female** | | | | | | | **Male** | | | | | | |
|  | **Ambient ozone pollution** | **Ambient particulate matter pollution** | **HAP from solid fuels** | **Low temperature** | **Occupational particulate matter, gases, and fumes** | **Secondhand smoke** | **Smoking** | **Ambient ozone pollution** | **Ambient particulate matter pollution** | **HAP from solid fuels** | **Low temperature** | **Occupational particulate matter, gases, and fumes** | **Secondhand smoke** | **Smoking** |
| **Gabon** | 2.0 | 21.9 | 2.2 | 0.6 | 6.7 | 4.7 | 6.5 | 2.6 | 22.4 | 1.5 | 0.7 | 12.3 | 3.7 | 38.8 |
| **Gambia** | 4.0 | 13.4 | 50.3 | 0.6 | 10.7 | 9.2 | 4.4 | 5.2 | 16.7 | 41.2 | 0.8 | 17.2 | 6.4 | 46.8 |
| **Ghana** | 5.2 | 21.4 | 22.9 | 0.2 | 18.7 | 3.1 | 6.1 | 7.7 | 24.3 | 17.0 | 0.3 | 21.9 | 2.5 | 28.0 |
| **Guinea** | 4.9 | 8.9 | 61.1 | 0.6 | 12.8 | 6.1 | 7.8 | 5.9 | 11.8 | 53.0 | 0.7 | 18.5 | 4.5 | 49.2 |
| **Guinea-Bissau** | 4.4 | 10.3 | 58.0 | 0.5 | 13.4 | 5.9 | 3.9 | 5.6 | 13.3 | 49.3 | 0.7 | 19.0 | 4.5 | 25.3 |
| **Kenya** | 3.1 | 8.0 | 37.9 | 4.6 | 16.0 | 4.2 | 10.1 | 3.7 | 9.3 | 31.2 | 5.6 | 20.6 | 3.1 | 42.5 |
| **Lesotho** | 4.5 | 12.1 | 29.0 | 14.8 | 10.6 | 10.6 | 15.0 | 5.3 | 14.0 | 21.9 | 17.5 | 18.3 | 6.2 | 62.6 |
| **Liberia** | 3.2 | 10.7 | 53.9 | 0.4 | 13.6 | 4.1 | 7.0 | 4.0 | 13.8 | 45.3 | 0.4 | 18.2 | 3.0 | 33.2 |
| **Madagascar** | 2.0 | 4.6 | 54.9 | 3.3 | 20.3 | 4.4 | 5.5 | 2.4 | 6.1 | 46.7 | 4.0 | 23.0 | 3.3 | 34.9 |
| **Malawi** | 2.8 | 5.0 | 58.1 | 3.3 | 17.0 | 4.0 | 11.0 | 3.6 | 6.7 | 50.7 | 4.1 | 16.8 | 3.0 | 49.8 |
| **Mali** | 4.5 | 8.3 | 67.7 | 2.6 | 13.9 | 5.3 | 6.6 | 5.5 | 11.1 | 59.5 | 3.2 | 19.2 | 3.9 | 39.1 |
| **Mauritania** | 2.8 | 24.0 | 24.1 | 2.5 | 10.0 | 5.5 | 9.8 | 3.5 | 26.9 | 17.8 | 3.2 | 14.0 | 4.0 | 42.3 |
| **Mauritius** | 0.0 | 9.3 | 0.3 | 1.2 | 7.6 | 12.2 | 10.6 | 0.0 | 9.6 | 0.2 | 1.4 | 16.7 | 7.1 | 58.6 |
| **Mozambique** | 2.0 | 3.8 | 63.8 | 1.7 | 18.6 | 4.0 | 11.2 | 2.8 | 5.2 | 57.5 | 2.3 | 19.3 | 2.9 | 42.9 |
| **Namibia** | 4.4 | 13.4 | 16.2 | 3.7 | 7.9 | 5.6 | 33.9 | 5.6 | 14.6 | 11.6 | 4.6 | 14.2 | 4.6 | 47.0 |
| **Niger** | 5.8 | 7.0 | 74.3 | 2.7 | 12.6 | 5.0 | 3.0 | 6.9 | 9.8 | 68.5 | 3.1 | 21.0 | 3.8 | 25.1 |
| **Nigeria** | 6.9 | 20.8 | 29.7 | 0.4 | 13.5 | 3.7 | 4.8 | 8.9 | 22.8 | 28.9 | 0.6 | 18.5 | 4.6 | 22.4 |
| **Rwanda** | 5.8 | 9.6 | 49.0 | 6.4 | 19.9 | 4.3 | 32.7 | 7.0 | 12.2 | 41.0 | 7.7 | 21.7 | 3.5 | 56.1 |
| **Sao Tome and Principe** | 3.6 | 13.4 | 28.3 | 0.3 | 6.1 | 2.3 | 8.4 | 4.3 | 15.4 | 21.2 | 0.4 | 15.2 | 1.9 | 32.1 |
| **Senegal** | 3.7 | 14.4 | 47.1 | 0.8 | 9.5 | 9.5 | 4.3 | 4.6 | 17.9 | 38.4 | 0.9 | 19.4 | 7.4 | 38.5 |
| **Seychelles** | 1.0 | 10.0 | 0.1 | 0.2 | 10.6 | 10.6 | 16.3 | 1.2 | 10.2 | 0.1 | 0.2 | 15.6 | 7.0 | 65.6 |
| **Sierra Leone** | 3.7 | 9.5 | 58.4 | 0.3 | 13.9 | 7.5 | 11.3 | 4.6 | 12.5 | 50.1 | 0.4 | 17.7 | 5.0 | 46.1 |
| **Somalia** | 2.9 | 1.9 | 83.5 | 1.0 | 14.5 | 4.7 | 8.4 | 3.6 | 2.9 | 80.7 | 1.2 | 21.9 | 3.7 | 43.4 |
| **South Africa** | 2.8 | 17.4 | 3.3 | 7.3 | 6.7 | 7.7 | 27.5 | 3.7 | 17.8 | 2.4 | 9.4 | 14.0 | 5.5 | 54.5 |
| **South Sudan** | 3.6 | 8.2 | 54.6 | 0.4 | 16.1 | 5.0 | 7.9 | 5.2 | 10.9 | 47.3 | 0.6 | 18.9 | 3.9 | 42.6 |
| **Sudan** | 5.5 | 22.5 | 19.0 | 2.4 | 5.6 | 10.3 | 9.5 | 6.6 | 24.9 | 13.8 | 2.9 | 17.3 | 9.3 | 57.0 |
| **Togo** | 6.5 | 12.2 | 46.6 | 0.3 | 17.1 | 5.6 | 13.3 | 8.3 | 15.3 | 38.1 | 0.4 | 19.8 | 4.5 | 45.8 |
| **Uganda** | 4.1 | 8.7 | 52.1 | 1.9 | 17.1 | 3.4 | 12.5 | 5.4 | 11.2 | 44.1 | 2.5 | 20.4 | 3.0 | 35.6 |
| **United Republic of Tanzania** | 2.5 | 6.6 | 50.5 | 2.8 | 17.4 | 6.2 | 19.6 | 3.3 | 8.7 | 43.0 | 3.5 | 21.3 | 4.1 | 50.0 |
| **Zambia** | 4.2 | 9.9 | 36.3 | 2.9 | 13.8 | 5.3 | 18.0 | 5.4 | 11.9 | 28.7 | 3.6 | 18.5 | 3.4 | 44.5 |
| **Zimbabwe** | 3.0 | 8.1 | 37.4 | 2.9 | 16.4 | 7.9 | 19.4 | 4.1 | 9.7 | 29.4 | 4.0 | 20.6 | 3.9 | 61.2 |

*Notes: Disability-adjusted life year; COPD - chronic obstructive pulmonary disease; HAP – household air pollution*
