# Supplementary material for: Burden and risk factors of chronic obstructive pulmonary disease in Sub-Saharan African countries, 1990–2019: a systematic analysis for the Global Burden of disease study 2019
Source: eClinicalMedicine. 2023 Oct 2;64:102215. doi: 10.1016/j.eclinm.2023.102215 (PMC10550520; doi:10.1016/j.eclinm.2023.102215)
Supplement: Appendix B [file mmc2.docx]

**Burden and risk factors of chronic obstructive pulmonary disease in Sub-Saharan African countries, 1990-2019: A systematic analysis for the Global Burden of Disease Study 2019**

Mulubirhan Assefa Alemayohu, PhD^1,2,3^ Maria Elisabetta Zanolin, PhD^1^ Lucia Cazzoletti, PhD^1^ Peter Nyasulu, PhD^4^ Vanessa Garcia-Larsen, PhD^5^ and ***GBD 2019 Sub-Saharan COPD Collaborators*^*^**

^1^ Unit of Epidemiology and Medical Statistics, University of Verona, Italy

^2^ School Public Health, Mekelle University, Ethiopia

^3^ Biostatistics and Clinical Epidemiology Unit, Department of Public Health, Experimental and Forensic Medicin, University of Pavia, 27100 Pavia, Italy

^4^ Faculty of Medicine and Health Sciences, Stellenbosch University, Cape Town, South Africa

^5^ Department of International Health, The Johns Hopkins Bloomberg School of Public Health, Baltimore, US

^*^ Authors list provided at the end of the manuscript.

## Authors’ Contributions

### Providing data or critical feedback on data sources

Yonas Derso Abtew, Denberu Eshetie Adane, Mulubirhan Assefa Alemayohu, Amadou Barrow, Belay Boda Abule Bodicha, Lucia Cazzoletti, Gashaw Sisay Chanie, Michael Ekholuenetale, Daniel Berhanie Enyew, Hawi Leul Esayas, Adeniyi Francis Fagbamigbe, Vanessa Garcia-Larsen, Segun Emmanuel Ibitoye, Olayinka Stephen Ilesanmi, Tesfaye K Kanko, Patrick D.M.C. Katoto, Shafiu Mohammed, Ogochukwu Janet Nzoputam, Chimezie Igwegbe Nzoputam, Osaretin Christabel Okonji, Mayowa O Owolabi, Yonatan Solomon, Gedif Ashebir Wubetie, Yazachew Yismaw, and Maria Eisabetta Zanolin.

### Developing methods or computational machinery

Mulubirhan Assefa Alemayohu, Amadou Barrow, Ogochukwu Janet Nzoputam, and Chimezie Igwegbe Nzoputam.

### Providing critical feedback on methods or results

Yonas Derso Abtew, Denberu Eshetie Adane, Miracle Ayomikun Adesina, Mulubirhan Assefa Alemayohu, Amadou Barrow, Alemshet Yirga Berhie, Belay Boda Abule Bodicha, Lucia Cazzoletti, Gashaw Sisay Chanie, Feleke Mekonnen Demeke, Lankamo Ena Digesa, Michael Ekholuenetale, Daniel Berhanie Enyew, Hawi Leul Esayas, Adeniyi Francis Fagbamigbe, Getahun Fetensa, Vanessa Garcia-Larsen, Kahsu Gebrekirstos Gebrekidan, Habtamu Alganeh Guadie, Segun Emmanuel Ibitoye, Olayinka Stephen Ilesanmi, Tesfaye K Kanko, Patrick D.M.C. Katoto, Samson Mideksa Legesse, Shafiu Mohammed, Jobert Richie Nansseu, Peter S Nyasulu, Ogochukwu Janet Nzoputam, Chimezie Igwegbe Nzoputam, Oluwakemi Ololade Odukoya, Osaretin Christabel Okonji, Mayowa O Owolabi, Bereket Beyene Shashamo, Yonatan Solomon, Worku Animaw Temesgen, Gedif Ashebir Wubetie, Yazachew Yismaw, Maria Eisabetta Zanolin, Getachew Assefa Zenebe.

### Drafting the work or revising it critically for important intellectual content

Denberu Eshetie Adane, Mulubirhan Assefa Alemayohu, Amadou Barrow, Belay Boda Abule Bodicha, Lucia Cazzoletti, Gashaw Sisay Chanie, Diriba Dereje, Daniel Berhanie Enyew, Adeniyi Francis Fagbamigbe, Getahun Fetensa, Vanessa Garcia-Larsen, Yibeltal Yismaw Gela, Segun Emmanuel Ibitoye, Olayinka Stephen Ilesanmi, Tesfaye K Kanko, Patrick D.M.C. Katoto, Samson Mideksa Legesse, Shafiu Mohammed, Jobert Richie Nansseu, Peter S Nyasulu, Ogochukwu Janet Nzoputam, Chimezie Igwegbe Nzoputam, Osaretin Christabel Okonji, Mayowa O Owolabi, Bereket Beyene Shashamo, Yonatan Solomon, Gedif Ashebir Wubetie, Maria Eisabetta Zanolin, Getachew Assefa Zenebe.

### Managing the estimation or publications process

Denberu Eshetie Adane, Mulubirhan Assefa Alemayohu, Belay Boda Abule Bodicha, Vanessa Garcia-Larsen, Peter S Nyasulu.
