# Supplementary material for: Burden and risk factors of chronic obstructive pulmonary disease in Sub-Saharan African countries, 1990–2019: a systematic analysis for the Global Burden of disease study 2019
Source: eClinicalMedicine. 2023 Oct 2;64:102215. doi: 10.1016/j.eclinm.2023.102215 (PMC10550520; doi:10.1016/j.eclinm.2023.102215)
Supplement: Supplementary Tables [file mmc3.docx]

**Suppl. Table 1** Number of input sources to estimate the burden and risk factors of chronic obstructive pulmonary disease in Sub-Saharan countries, 1990-2019.

| **Categories** | **Component(s)** | **No of distinct datapoints^[[1]](#footnote-1)^** |
| --- | --- | --- |
| **Cause of death and disability** | Nonfatal Health Outcomes | 7 |
|  | Causes of Death | 16 |
| **Risk factors** | Household air pollution from solid fuels | 393 |
|  | Ambient particulate matter pollution | 28 |
|  | Ambient ozone pollution | 0^[[2]](#footnote-2)^ |
|  | Low temperature | 1 |
|  | Occupational particulate matter, gases, and fumes, | 81 |
|  | Smoking | 285 |
|  | Secondhand smoke | 192 |
| **Mortality and Population** | Population | 243 |

1. <https://ghdx.healthdata.org/gbd-2019/data-input-sources?components=9&locations=166> [↑](#footnote-ref-1)
2. There is no datapoint available and it was obtained using simulation modelling of ozone ground measurement data from the Tropospheric Ozone Assessment Report (TOAR) [↑](#footnote-ref-2)
