# Supplementary material for: Burden and risk factors of chronic obstructive pulmonary disease in Sub-Saharan African countries, 1990–2019: a systematic analysis for the Global Burden of disease study 2019
Source: eClinicalMedicine. 2023 Oct 2;64:102215. doi: 10.1016/j.eclinm.2023.102215 (PMC10550520; doi:10.1016/j.eclinm.2023.102215)
Supplement: PubMed SSA COPD [file mmc4.docx]

# GBD 2019 Sub-Saharan Africa COPD Collaborators

| **First Name** | **Last Name** |
| --- | --- |
| Mulubirhan Assefa | Alemayohu* |
| Maria Eisabetta | Zanolin^+^ |
| Lucia | Cazzoletti |
| Peter S | Nyasulu |
| Vanessa | Garcia-Larsen |
| Yonas Derso | Abtew |
| Denberu Eshetie | Adane |
| Miracle Ayomikun | Adesina |
| Amadou | Barrow |
| Alemshet Yirga | Berhie |
| Belay Boda Abule | Bodicha |
| Gashaw Sisay | Chanie |
| Feleke Mekonnen | Demeke |
| Diriba | Dereje |
| Lankamo Ena | Digesa |
| Michael | Ekholuenetale |
| Daniel Berhanie | Enyew |
| Hawi Leul | Esayas |
| Adeniyi Francis | Fagbamigbe |
| Getahun | Fetensa |
| Kahsu Gebrekirstos | Gebrekidan |
| Yibeltal Yismaw | Gela |
| Habtamu Alganeh | Guadie |
| Segun Emmanuel | Ibitoye |
| Olayinka Stephen | Ilesanmi |
| Tesfaye K | Kanko |
| Patrick D.M.C. | Katoto |
| Samson Mideksa | Legesse |
| Shafiu | Mohammed |
| Jobert Richie | Nansseu |
| Ogochukwu Janet | Nzoputam |
| Chimezie Igwegbe | Nzoputam |
| Oluwakemi Ololade | Odukoya |
| Osaretin Christabel | Okonji |
| Mayowa O | Owolabi |
| Bereket Beyene | Shashamo |
| Yonatan | Solomon |
| Worku Animaw | Temesgen |
| Gedif Ashebir | Wubetie |
| Yazachew | Yismaw |
| Getachew Assefa | Zenebe |

*Lead author
^+^Senior author
